# Cytoarchitectonic mapping of the human frontal operculum—New correlates for a variety of brain functions

**DOI:** 10.3389/fnhum.2023.1087026

**Published:** 2023-06-28

**Authors:** Nina Unger, Martina Haeck, Simon B. Eickhoff, Julia A. Camilleri, Timo Dickscheid, Hartmut Mohlberg, Sebastian Bludau, Svenja Caspers, Katrin Amunts

**Affiliations:** ^1^Cécile and Oskar Vogt Institute for Brain Research, Medical Faculty and University Hospital Düsseldorf, Heinrich Heine University Düsseldorf, Düsseldorf, Germany; ^2^Institute of Neuroscience and Medicine (INM-1), Research Centre Jülich, Jülich, Germany; ^3^MEDIAN Klinik Wismar, Wismar, Germany; ^4^Institute of Neuroscience and Medicine (INM-7), Research Centre Jülich, Jülich, Germany; ^5^Institute for Systems Neuroscience, Medical Faculty and University Hospital Düsseldorf, Heinrich Heine University Düsseldorf, Düsseldorf, Germany; ^6^Institute of Computer Science, Heinrich Heine University Düsseldorf, Düsseldorf, Germany; ^7^Institute for Anatomy I, Medical Faculty and University Hospital Düsseldorf, Heinrich Heine University Düsseldorf, Düsseldorf, Germany

**Keywords:** Julich-Brain Atlas, BigBrain, cerebral cortex, cytoarchitecture, language, music, frontal operculum, probability maps

## Abstract

The human frontal operculum (FOp) is a brain region that covers parts of the ventral frontal cortex next to the insula. Functional imaging studies showed activations in this region in tasks related to language, somatosensory, and cognitive functions. While the precise cytoarchitectonic areas that correlate to these processes have not yet been revealed, earlier receptorarchitectonic analysis resulted in a detailed parcellation of the FOp. We complemented this analysis by a cytoarchitectonic study of a sample of ten postmortem brains and mapped the posterior FOp in serial, cell-body stained histological sections using image analysis and multivariate statistics. Three new areas were identified: Op5 represents the most posterior area, followed by Op6 and the most anterior region Op7. Areas Op5-Op7 approach the insula, up to the circular sulcus. Area 44 of Broca’s region, the most ventral part of premotor area 6, and parts of the parietal operculum are dorso-laterally adjacent to Op5-Op7. The areas did not show any interhemispheric or sex differences. Three-dimensional probability maps and a maximum probability map were generated in stereotaxic space, and then used, in a first proof-of-concept-study, for functional decoding and analysis of structural and functional connectivity. Functional decoding revealed different profiles of cytoarchitectonically identified Op5-Op7. While left Op6 was active in music cognition, right Op5 was involved in chewing/swallowing and sexual processing. Both areas showed activation during the exercise of isometric force in muscles. An involvement in the coordination of flexion/extension could be shown for the right Op6. Meta-analytic connectivity modeling revealed various functional connections of the FOp areas within motor and somatosensory networks, with the most evident connection with the music/language network for Op6 left. The new cytoarchitectonic maps are part of Julich-Brain, and publicly available to serve as a basis for future analyses of structural-functional relationships in this region.

## 1. Introduction

Broca’s and Wernicke’s regions are considered to be key regions of language processing (Grodzinsky and Friederici, 2006; Grodzinsky and Santi, 2008; Friederici, 2011; DeWitt and Rauschecker, 2013; Binder, 2017; Friederici, 2017). Broca’s region with areas 44 and 45 (Amunts et al., 1999, 2004) can be found in the left inferior frontal gyrus (IFG) and is part of the anterior language region. Wernicke’s region (including posterior part of area 22) (Morosan et al., 2005) is located in the left superior temporal gyrus and is part of the posterior language region (Amunts and Zilles, 2012). Broca’s and Wernicke’s regions are connected through the arcuate fascicle and the left superior longitudinal fascicle (dorsal pathway II) as well as through the extreme capsule fiber system (ventral pathway I) [Catani et al., 2005; Friederici, 2009, 2011, 2012; Fernández-Miranda et al., 2015, for reviews see Bernal and Ardila (2009) and Bernard et al. (2019)].

Language networks comprise a large number of brain regions [for a review see Hagoort, 2014]. It has been emphasized that Broca’s region is heterogeneous and consists of multiple, functionally distinct components (Hagoort, 2019; Fedorenko and Blank, 2020). The hypothesis was formulated that the dorsal language system, which includes area 44 and the posterior part of the superior temporal gyrus is complemented by the ventral language system, consisting of area 45 and the anterior part of the superior temporal gyrus (Skeide et al., 2016). Moreover, the anterior language region does not only consist of Broca’s region in a narrow sense, but may include neighboring areas of the ventral frontal cortex (Hagoort, 2014). These findings can be interpreted in the sense that the classical Broca and Wernicke regions might be anatomically ill-defined and should be replaced by more accurate descriptions (Tremblay and Dick, 2016). This also implies, however, a need to study the anatomical neighbors of classically defined Broca’s and Wernicke’s regions with respect to their structure and putative function in language processing.

Research from our own group has proposed a new segregation of the broader anterior language region that goes beyond classical areas 44 and 45 (Amunts et al., 2010). Using quantitative receptor autoradiography of major neurotransmitter receptors, areas 44 and 45 and neighboring areas including caudally adjacent motor/premotor areas and ventrally adjacent ventral frontal cortex have been analyzed. Areas 44 and 45 have been further subdivided (each into two areas), and new areas in the frontal operculum (FOp) and premotor cortex have been identified (Amunts et al., 2010; Zilles and Amunts, 2018). Recent maps of areas Op8 and Op9 of the FOp (Saal et al., 2021a,b), which are located rostro-ventrally to areas 44 and 45 have confirmed this concept. These areas show a specific receptor architecture, distinct from classical language areas 44 and 45. Along the same line, connectivity-based analysis and probabilistic fiber tracking showed a subdivision of areas 44 and 45, and the deep FOp (Anwander et al., 2007; Jung et al., 2017). The FOp is connected with the anterior part of the superior temporal gyrus and the superior temporal sulcus via the uncinate fascicle (ventral pathway II) (Catani et al., 2005; Friederici, 2009, 2011). Furthermore, areas of the FOp seem to be phylogenetically older than areas 44 and 45 (Friederici, 2006), which is also in line with the assumption that the FOp region is functionally distinct.

The FOp has been discussed in the context of language: It seems to be mainly involved in the processing of phonological and syntactical information, both in healthy subjects and in patients with language disorders. For phonological processing, a stronger blood-oxygen-level-dependent response in the FOp was reported during auditory rhyme processing (rhymed trials) compared to non-rhymed trials (Huschler et al., 2013). In case of syllable sequence production, the FOp showed an increased activation for stimuli with a higher phonological complexity (Bohland and Guenther, 2006). Patients with left FOp lesions were found to perform less accurately in reading non-words and comparable phonological tasks (Fiez et al., 2006). A functional magnetic resonance imaging (fMRI) study (Friederici et al., 2000) reported an increased activity in the lower tip of the left FOp during a syntactic judgment task (deciding whether a stimulus belongs to nouns or to function words, e.g., conjunction or preposition) compared with a semantic judgment task (deciding whether a stimulus belongs to concrete or abstract words). Here, non-prototypical members of word types (concrete function words, e.g., because or if as well as abstract nouns, e.g., pain) were reported to show higher activation and neural resources compared with prototypical word type members (concrete nouns, e.g., house as well as abstract function words, e.g., on or above) (Friederici et al., 2000). Another fMRI experiment on syntax reported local specific activations in the left FOp during a task of transition processing, i.e., the evaluation concerning transitional dependencies (Friederici et al., 2006a). An event-related fMRI study demonstrated that processing of sentences with ungrammatical word orders lead to a selective engagement of the more posterior deep FOp (Friederici et al., 2006b). In addition to its involvement in language, the FOp seems to play a role for gustatory representation, task control, and the perception of visceral stimuli (Eickhoff et al., 2006b; Higo et al., 2011; Veldhuizen et al., 2011; Chikazoe et al., 2019; Quirmbach and Limanowski, 2022) as well as executive control [(Thompson-Schill et al., 1997; Novick et al., 2005), for a review see Gratton et al. (2018)], according to activation patterns reported for this region. It would be plausible to assume that such diversity of function is not supported by a single brain region, but by several.

Some language processing functions have been shown to differ between genders. For example, in terms of rhyming, an echo-planar fMRI study showed a lateralized activity in the left IFG in male participants, but not females. In contrast, the pattern of activation in females showed more diffuse involvement of the left and right IFG, which was interpreted by the authors as evidence for organizational differences between genders in phonological processing (Shaywitz et al., 1995). To our knowledge, existing studies on gender differences in language mostly consider Broca’s region, while neighboring regions, e.g., FOp, have not been addressed. However, the structural correlates of the differences and whether they also affect the FOp are largely unknown.

**FIGURE 1:**
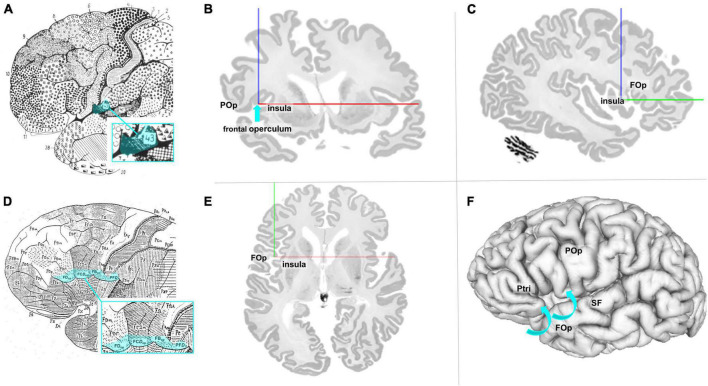
**(A)** Modified cytoarchitectonic map of Brodmann (1909), including BA 43 (blue highlighted). Details of the cortex within the sulci or areas ventrally to areas 44, 45, and 47 were not shown by Brodmann. Area 43 (highlighted in blue) is located slightly posterior to the referred area. **(B)** Coronal section of the BigBrain (Amunts et al., 2013). The blue arrow points to the macroanatomic location of lefthemispheric FOp. POp, parietal operculum. **(C)** Sagittal section of the BigBrain with annotations of the FOp and the insula. **(D)** Modified cytoarchitectonic mapping of von Economo and Koskinas (1925) with highlighted areas FD_op_, FC*D*_op_, FB_op_, and PF*D*, localized at the ventral end of the frontal lobe. **(E)** Axial section of the BigBrain with annotations of the FOp and the insula. **(F)** View of the left surface of the BigBrain. The blue arrows show that the FOp is also found below the brain surface. POp, parietal operculum; Ptri, triangular part of the inferior frontal gyrus; SF, Silvian fissure. Physical coordinates (cursor) for panels **(B,C,E,F)**: –38.637 mm/22.526 mm/0.444 mm (https://atlases.ebrains.eu/viewer).

The FOp and its cytoarchitectonic parcellation have not been addressed so far. The map of Brodmann (1909) showed Brodmann’s area (BA) 43 as the posterior neighbor of Broca’s region; it is located at the inferior edge of the postcental gyrus and superior to the lateral fissure as well as in the cingulate region of the cortex (see Figure 1A). The Brodmann map, however, does not show any details of the cortex within the sulci or any parcellation details ventrally to BA 44, BA 45, and BA 47. The cytoarchitectonic map of von Economo and Koskinas (1925) described the subcentral area PF*D*, area FB_op_ of the frontal lobe, the opercular intermediate frontal area FC*D*_op_, and area FD_op_ as possible anatomical correlates for the posterior part of the FOp (Figure 1D). Nevertheless, both maps share the problem that they represent 2D schematic drawings of the lateral surface of the brain, but do not well disclose cortical areas that are obscured by the bulging cortex of the (lateral) frontal cortex. More recent maps of Petrides and Pandya (1994), designated the area anterior to the central sulcus as area 6 (6VR and 6VC). Öngür et al. (2003) described a precentral opercular area (PrCO) adjacent to the insular cortex and located in the inside of the lateral fissure representing parts of the FOp. A more detailed view into the complex 3D geometry of the FOp is necessary to study the spatial relationships of the IFG, the FOp, and the neighboring insula. This became possible in the BigBrain model, a histological representation of cell body stained sections at 20 μm isotropic spatial resolution (Amunts et al., 2013; see Figures 1B, C, E, F).

Because of these open questions and the lack of precise microstructural maps that would be instrumental in further developing the concept of an anterior language region, the aim of the study was (a) to analyze the posterior part of the FOp and its segregation, (b) to generate 3D maps based on cytoarchitectonic mapping in a sample of ten brains to take into account variations in anatomy between brains (Amunts and Zilles, 2015), (c) to evaluate potential interhemispheric and gender differences, and (d) to perform meta-analytic connectivity modeling (MACM) using the BrainMap database (Laird et al., 2009; Robinson et al., 2010), considering complementary connectivity data, to gain further insights into the functions of the areas.

## 2. Materials and methods

### 2.1. Histological and image processing

Ten human postmortem brains were analyzed (five females and five males; Supplementary Table 1), which were obtained via the body donor program of the Anatomical Institute of the Heinrich Heine University Düsseldorf. Written informed consent from body donors was obtained. The donors had no clinical history of neurological or psychiatric diseases. The study was performed according to the guidelines of the Ethics Committee of the University of Düsseldorf (#4863).

The brains were extracted within less than 24 h after death and fixed in 4% formalin or in Bodian’s fixative for at least 6 months, and further processed. A MRI scan of each brain was obtained using a 1.5-T Siemens scanner (Erlangen, Germany) using a T1-weighted 3D FLASH sequence (flip angle: 40°, repetition time: 40 ms, time echo: 5 ms). The scans served as undistorted references for subsequent 3D reconstruction. The brains were then embedded in paraffin and serially sectioned in the coronal plane (Polycut E, Reichert-Jung, Germany; thickness = 20 μm). Each 15th section (distance between mounted sections: 300 μm) was stained for cell bodies (Merker, 1983). The whole procedure and subsequent mapping, cytoarchitectonic analysis, and the computation of cytoarchitectonic probability maps (see sections 2.1–2.5) is in detail described in Amunts et al. (2020).

### 2.2. Observer-independent identification of cytoarchitectonic borders based on the gray level index (GLI)

**FIGURE 2:**
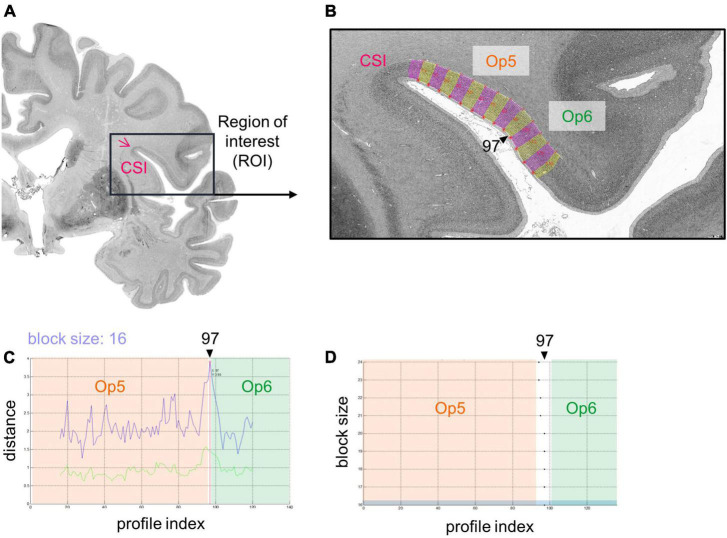
Analysis of a cytoarchitectonic border between Op5 and Op6, right hemisphere using an observer-independent mapping approach (Schleicher et al., 1999). **(A)** A region of interest (ROI) was identified (black box), which covered the whole extent of the FOp including the circular sulcus of the insula (CSI). It was scanned in a mosaic like pattern. Measurements were transformed into a gray level index (GLI) image that showed the volume fraction of cell bodies. **(B)** Equidistant traverses perpendicular to the outer and inner contour line were calculated (pink and yellow lines, changing after every 10th traverse). GLI profiles along these traverses displayed laminar changes in cytoarchitecture. CSI, circular sulcus of the insula. **(C)** The Mahalanobis distance (MD) (Mahalanobis et al., 1949) was used as a measure to quantify dissimilarity in shape of GLI profiles between neighboring areas (see upper blue curve; lower green curve: Euclidean distance function) (Schleicher et al., 2000). By means of a sliding window technique, profiles with different block sizes were moved along the cortical ribbon. For the specific block size 16, MD reaches a significant local maximum at the border between areas Op5 and Op6, at profile position 97. **(D)** Resulting significant maxima of the MD function showed profile positions with potential cytoarchitectonic borders (in the example, at profile position 97). In case MD resulted in significant maxima for different block sizes at similar profile positions [Blue marked block size: Block size 16 from panel **(C)**] and in case this border was found in three comparable positions in a minimum of three adjacent brain sections, a peak was accepted as a cytoarchitectonical border.

Cytoarchitectonic borders between areas of the FOp were identified using image analysis and statistical tools (Schleicher et al., 1999, 2005, 2009). Hereby, rectangular regions of interest (ROIs) (Figure 2A) were identified in the images of the histological sections, and scanned in a mosaic like pattern. For this purpose, a high-resolution CCD-Camera (Axiocam MRm, ZEISS, Germany) was used, combined with an optical light microscope (Axioplan 2 imaging, ZEISS, Germany). ROIs of every 60*^th^* histological section were digitized with an in-plane resolution of 1.02 μm per pixel in both hemispheres with ZEISS image analysis software Axiovision (Version 4.6, ZEISS, Germany). Digitized ROIs were transformed into gray level index (GLI) images using an in-house software written in Matlab (The MathWorks, Inc., Natick, MA, USA) (Schleicher et al., 2005, 2009; Bludau et al., 2014). The intensities of the pixels of a GLI image correspond to the volume fraction of stained cell bodies, ranging from 0% to 100% (Wree et al., 1982; Schleicher et al., 2000).

Outer (border between layers I and II) and inner contour lines (border between layer VI and the white matter) were drawn manually in each GLI image using Matlab scripts. Equidistant traverses were generated perpendicular to the two contour lines (Figure 2B; Schleicher et al., 1999, 2000, 2009). GLI profiles running along these curvilinear traverses quantified laminar changes in the volume fraction of cell bodies, i.e., the regional cytoarchitecture (Schleicher et al., 2009). A feature vector with ten elements was calculated including the mean GLI, center of gravity, standard deviation, skewness, and kurtosis of the original profile, and the same features of its first derivative. Individual and local variations of cortical thickness were normalized by length normalization to compensate for differences in thickness of the cortex between brains, areas, and geometry (Schleicher et al., 1999).

The Mahalanobis distance (MD) (Mahalanobis et al., 1949) was used to quantify dissimilarities in the shape of mean GLI profiles between neighboring blocks of profiles as measures of cytoarchitectonic differences between brain areas (Schleicher et al., 2005). To increase the robustness of the MD calculation, a number of profiles (range: 10–24 profiles) was grouped into blocks of profiles. Afterward, the MD between adjacent blocks was calculated. A sliding window technique was used to systematically move the different blocks along the cortical ribbon of the ROI. The profile blocks were shifted by one profile at a time (Schleicher et al., 1999, 2005). The larger the difference in shape between profiles of adjacent blocks, the larger was their MD.

A significant maximum of the Mahalanobis distance (Bonferroni-corrected Hotelling’s T2 test) indicated a cytoarchitectonic border between two adjacent cortical areas (Figure 2C). A border was accepted if the MD resulted in a significant maximum for different block sizes (Figure 2D) and the border was found at comparable positions in three or more adjacent brain sections. Subsequently, each border passed a quality control to exclude “artificial borders,” e.g., due to blood vessels, wrinkles, or other artifacts (Schleicher et al., 1999).

### 2.3. Hierarchical cluster analysis of area-specific cytoarchitecture

By performing a Matlab-based hierarchical cluster analysis, we aimed to determine the degree of cytoarchitectonic similarities and/or dissimilarities between FOp areas compared with areas 44 and 45 (Amunts et al., 1999, 2004). For each area, 10–15 consecutive GLI profiles were selected, coming from three sections per hemisphere and brain. Profiles from the left and right hemispheres of Op5-Op7 were merged in the present study, since no volumetric differences between left and right hemispheres were found (see section 3.4). For each profile, a ten-element feature vector was calculated to enable quantification of similarity/dissimilarity between the different areas (Schleicher et al., 2005). Hierarchical clustering of these feature vectors was performed using the Euclidean distance and the Ward linkage method (Ward, 1963). Cytoarchitectonic similarity between two areas was reflected by a low Euclidean distance, whereas cytoarchitectonic dissimilarity was associated with a higher Euclidean distance. The results were visualized in a hierarchical dendrogram (Figure 5).

### 2.4. 3D cytoarchitectonic probability maps in stereotaxic reference space and maximum probability maps (MPMs)

Delineations of FOp areas were manually transferred onto corresponding digitized sections (1,200 dpi high-resolution). Afterward, they were 3D reconstructed in each of the 10 postmortem brains. Images of the brains and their areas were then registered to the T1-weighted single-subject brain Colin27 of the MNI reference space and the ICBM152casym space (Holmes et al., 1998; Evans et al., 2012). The identified areas were superimposed in the two reference spaces, and 3D continuous probabilistic maps were generated (Amunts et al., 2020). Briefly, after calculating a linear affine transformation [7 degrees of freedom (DOF): 3 translations, 3 rotations, 1 isotropic scaling] as well as a non-linear transformation vector field (3 translations for every voxel of the data set) with optimized parameters for the MRI signal of the *in vivo* reference data set and the 3D reconstructed histologic postmortem brain data, the transformations of histological data sets were stored and then applied to the mapped cytoarchitectonic regions per set of reconstructed postmortem data. For this purpose, the calculated transformations for the whole brain were applied to all regions mapped in the 10 postmortem brains and superimposed in the reference space. Volume files were used to store the resulting probabilistic cytoarchitectonic maps. In the next step, the probabilistic values were projected onto a cortical surface model of the reference brain, computed with FreeSurfer, and a surface-based representation of the cytoarchitectonic maps was computed (Amunts et al., 2020). The maps showed, for each voxel of the reference brain, the probability that a cortical area was located at a given position of the reference space. Thereby, intersubject anatomical variability of cortical areas was quantified. Subsequently, a maximum probability map (MPM) was calculated (Eickhoff et al., 2005), which assigned each voxel of the reference brain to the cortical area with the highest probability in the respective voxel.

### 2.5. Volumetric analysis

The volumes of each FOp area were calculated per hemisphere and brain, considering the individual shrinkage-corrected volume for each brain and area. Beforehand, shrinkage factors were calculated as the ratio between the brain volume before and after the histological processing procedure (Amunts et al., 2020). In order to compare volumes of areas from brains with different size, the volume of each area was additionally normalized using the individual total brain volume (normalized volume = shrinkage-corrected individual area volume/shrinkage-corrected individual whole-brain volume). Thus, relative volume ratios were used for volume calculation to allow comparability between different brains.

The shrinkage corrected and normalized mean volumes for the different areas were tested for hemisphere and gender differences using a non-parametric pair-wise permutation test. The null distribution was evaluated with Monte Carlo simulation (1,000,000 iterations). Differences were considered to be significant if they were larger than 95% of values under the null-hypothesis [*p* < 0.05, false discovery rate (FDR) corrected for multiple comparisons] (Eickhoff et al., 2007; Bludau et al., 2014).

### 2.6. Functional decoding of areas Op5, Op6, and Op7

To functionally characterize areas Op5-Op7, we used a MACM based analysis on the BrainMap database^1^ (Fox and Lancaster, 2002; Laird et al., 2005). MPMs of left and right areas Op5, Op6, and Op7 in MNI ICBM 152 reference space were defined as volumes of interest (VOIs). The BrainMap database contains 3D coordinates as results from published task-related neuroimaging experiments combined with meta-data describing “behavioral domains” and “paradigm classes” (Laird et al., 2009).

The following BrainMap Sleuth search criteria were used to filter for matching functional imaging studies with 3D peak coordinates: At the level of imaging modality, fMRI and positron emission tomography data of healthy subjects were considered, with at least one activation focus. Deactivations were excluded. Only normal mapping studies (no interventions and no group comparison) were considered (Laird et al., 2011). The search criteria led to a total number of 2,620 matching functional neuroimaging experiments at the time of analysis for further evaluation. No preselection of taxonomic categories was applied.

Analysis of meta-data involved the acquisition of all detectable functions by the over-representation of behavioral domains and paradigm classes in the experiments activating each VOI relative to the BrainMap database (Eickhoff et al., 2011). Whereas behavioral domains refer to mental processes of six neural main categories and related subcategories (cognition, action, perception, emotion, interoception, pharmacology), paradigm classes refer to single experimental tasks [(Laird et al., 2009), available online, http://www.brainmap.org/taxonomy/]. Bar charts visualized activated behavioral domains and paradigm classes for each examined VOI with its respective probability likelihood ratio. For this, forward and reverse inference were applied. Forward inference describes the probability of observing activity in a brain region, taking into account knowledge of a predefined behavioral domain or paradigm class. Reverse inference describes the probability of a particular behavioral domain or paradigm class, given activation in a VOI. Significance was established with a binomial test [*p* < 0.05, FDR corrected for multiple comparisons] (Laird et al., 2009; Eickhoff et al., 2011).

### 2.7. Functional and structural connectivity of areas Op5, Op6, and Op7

In a first step, we integrated functional connectivity using MACM, and aimed to analyze whole-brain co-activation patterns of Op5-Op7 left/right. Therefore, MACM analyses were performed using the activation likelihood estimation algorithm. To analyze the functional connectivity (co-activation) of each FOp area, concordant activation foci were determined per area from reported neuroimaging activation data obtained from BrainMap (Eickhoff et al., 2011). Due to spatial uncertainty of neuroimaging data, we modeled the foci as probability distributions. As a result, MACM generated coincided whole-brain co-activation maps for each FOp area (peak x-y-z coordinates in stereotaxic reference space). They were displayed on the MNI ICBM 152 brain template. To define a null-distribution reflecting a random spatial assignment of experiments, a permutation test with 10,000 repetitions was used (*p* < 0.05, FDR corrected) (Eickhoff et al., 2016). To analyze equal and differing co-activation patterns of Op5–Op7, we carried out a conjunction analysis and a contrast analysis (Nichols et al., 2005). The conjunction analysis is based on identifying brain areas that are activated by task A and task B or combined tasks (Price and Friston, 1997; Nichols et al., 2005). In further application, it included overlap of the six single MACM co-activation maps and enabled us to investigate which brain regions demonstrated co-activation with all three opercular areas Op5, Op6, and Op7, or combinations of the areas. The contrast analysis was performed by voxel-wise calculation of differences between co-activation maps of individual MACM analyses. The analysis was conducted to demonstrate differing functional connectivity between two respective VOIs as well as to highlight unique functions for each area per hemisphere (Eickhoff et al., 2011).

In a second step, we studied the structural connectivity of bilateral areas Op5, Op6, and Op7 using connectivity data of the population-based German cohort study 1000BRAINS (Caspers et al., 2014). Connectivity data can be accessed online via 3D atlas viewer “siibra-explorer” of the Human Brain Project’s EBRAINS research infrastructure.^2^ In the 1000BRAINS study, structural connectivity was analyzed on the basis of diffusion-weighted MRI obtained in a 3T MR scanner (sequence parameters of two datasets: b = 1,000 s/mm^2^ and 60 diffusion-weighted volumes; b = 2700 s/mm^2^ and 120 diffusion-weighted volumes, both at an isotropic resolution of 2.4 mm) (Caspers and Schreiber, 2021). Whole-brain connectivity matrices based on brain regions included in the Julich-Brain cytoarchitectonic maps were used as representations of the variability of fiber tracts, i.e., structural connectivity, in the 1000BRAINS population sample (Caspers et al., 2014).

## 3. Results

### 3.1. Cytoarchitecture of areas Op5–Op7 and distinction from neighboring areas

Three new cytoarchitectonic areas were identified, which differed in their microstructure. Op5 showed a prominent internal granular layer IV. Layer IV was thinner in areas Op6 and Op7. I.e., area Op5 is a granular area, while Op6 and Op7 are dysgranular. Details about cytoarchitectonic criteria for Op5, Op6, and Op7 can be found in Table 1.

**TABLE 1:** Cytoarchitectonic characteristics of opercular areas Op5, Op6, and Op7.

Area	Cytoarchitectonic characteristics
Op5	• Rather granular cytoarchitectonic area • Distinct horizontal lamination • Gradient in pyramidal cell size of layer III • Large pyramids in layers III and V • Broad layer IV with a high cell density and well defined borders to layers III and V • Large-sized pyramidal cells in layers IIIc and V adjusting directly to layer IV • High cell density in layer VI
Op6	• Dysgranular cytoarchitectonic area • Lower overall cell density compared with areas Op5 and Op7 • Poor cell density apart from layers IIIc and IV • Arrangement of cells mainly in columns • Border between layer II and layer III clear cut due to a high amount of cells in layer II • Layer IV not clearly visible due to intermingling with pyramidal cells from layers III and V • Prominent large sized and elongated pyramidal cells in layer V • Thick layer VI with numerous small cells
Op7	• Dysgranular cytoarchitectonic area • High cell density of layer IIIc, layer IV and the upper layer V • Lower cell density of layers IIIa + b and the lower layer V of Op7 • Large-sized pyramidal cells within the lower layer III and layer V • Blurred transition with the white matter, mainly because of the low cell density in the lower portion of layer VI

**FIGURE 3:**
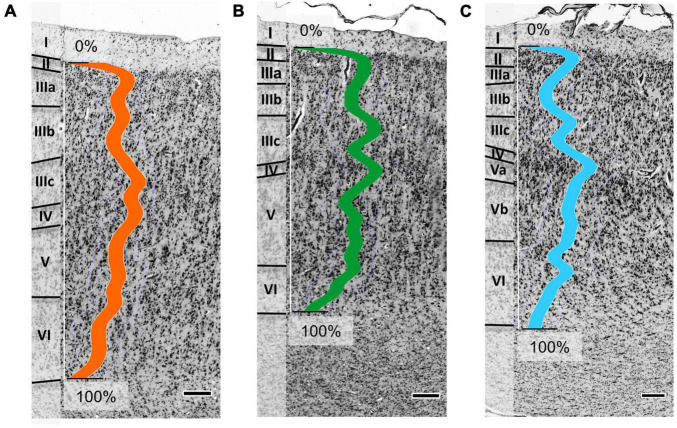
Cytoarchitecture of areas Op5 **(A)**, Op6 **(B)**, and Op7 **(C)** (Op5 and Op6 taken from brain BC20, right hemisphere; Op7 taken from brain BC21, right hemisphere). The colored lines indicate the mean gray level index (GLI) profiles of areas Op5 (orange line), Op6 (green line), and Op7 (blue line) for a range from 0% (outer contour line, border between layers I and II) and 100% (inner contour line, border between layer VI and the white matter). Roman numbers indicate the cortical layers. Scale bars = 200 μm.

Area Op5 showed a rather prominent laminar pattern (Figure 3A). Layer III revealed a gradient in pyramidal cell size from superficial (smaller cells) to deep parts (larger cells), with largest cell size in the lower layer III. In addition, Op5 showed large pyramidal cells in layer V. Layer IV was broad and densely packed, with a higher cell density than in layer V. The borders of layer IV to layers III and V were well defined.

Area Op6 was distinct from Op5 and Op7 by a somewhat lower overall cell density (Figure 3B). Op6 showed more pronounced vertical columns than the other two areas. Layer II showed a high cell density, and its border to layer III was clearly visible. Layer IV was intermingled by pyramidal cells from layers III and V. Prominent and elongated pyramidal cells were found in layer V. Layers IIIc and IV had a higher cell density than layer V. Layer VI was broad, with numerous small cells.

Area Op7 differed from Op5 and Op6 by its densely packed layers IIIc, IV, and the upper layer V (Figure 3C). In contrast, layers IIIa+b and lower layer V of Op7 showed a lower cell density. Large pyramidal cells were found in lower layer III and layer V. The transition to the white matter was blurred, mainly because of the low cell density in the lower portion of layer VI.

**FIGURE 4:**
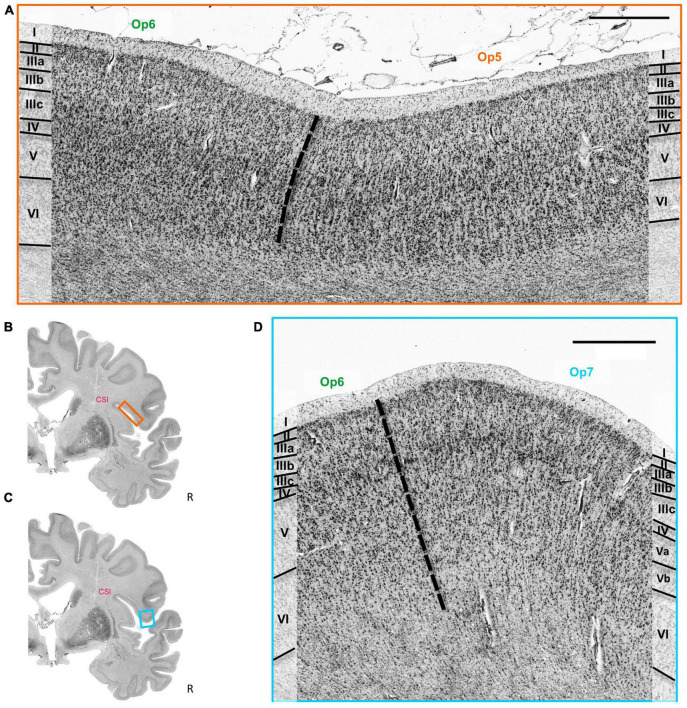
**(A)** Cytoarchitectonic border (dashed line) between the areas Op5 and Op6. Roman numbers indicate cortical layers. CSI = circular sulcus of the insula. Scale bar = 1000 μm. **(B)** Localization of the brain section in panel **(A)**. **(C)** Localization of the brain section in panel **(D)**. **(D)** Cytoarchitectonic border (dashed line) between the areas Op6 and Op7. Roman numbers indicate the cortical layers. CSI, circular sulcus of the insula. Scale bar = 1000 μm.

The cytoarchitectonic differences between the three Op-areas and neighboring areas were captured by the observer-independent mapping procedure, and the localization of borders were proven by multivariate statistics (see section 2). Exemplary cytoarchitectonic borders between areas Op5 and Op6 are shown in Figures 4A, B, and between Op6 and Op7 in Figures 4C, D.

**FIGURE 5:**
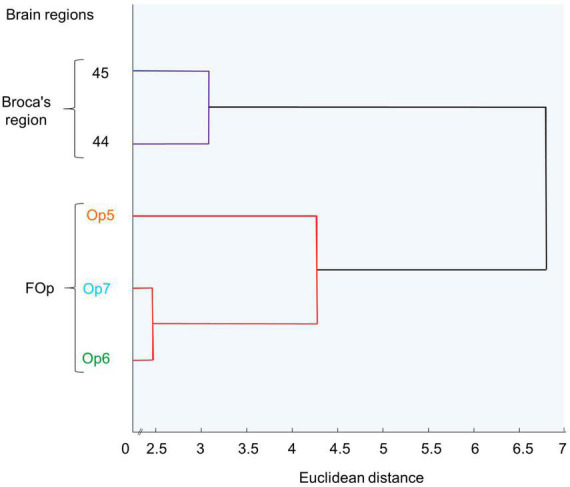
Hierarchical cluster analysis of areas Op5, Op6, Op7, and areas 44 and 45. Op6 and Op7 are similar to each other and form a cluster, distinct from Op5. Areas 44 and 45 form a distinct cluster.

A cluster analysis (Figure 5) further quantified similarities and dissimilarities between the areas. It showed that areas Op6 and Op7 as the two dysgranular areas were most similar to each other, while area Op5 as granular area was distinct. In the diagram, areas 44 and 45 were separated from the three FOp areas, and located on another branch of the hierarchical cluster tree. This indicates a greater cytoarchitectonic difference between areas 44 and 45 to the FOp than within Op5–Op7.

**FIGURE 6:**
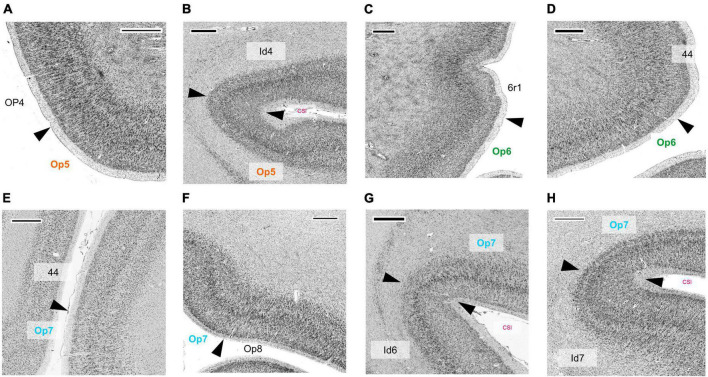
Cytoarchitectonic borders between areas Op5 and OP4 (Eickhoff et al., 2006a,c) **(A)**, between area Op5 and insular area Id4 (Quabs et al., 2021a) **(B)**, between area Op6 and premotor area 6r1, which is not yet mapped **(C)**, between area Op6 and area 44 (Amunts et al., 1999, 2004) **(D)**, between area Op7 and area 44 **(E)**, between areas Op7 and Op8 (Saal et al., 2021a) **(F)**, between areas Op7 and Id6 (Quabs et al., 2021b) **(G)**, and between area Op7 and Id7 (Grodzinsky et al., 2020) **(H)**. CSI, circular sulcus of the insula. Scale bars = 1,000 μm.

In comparison, the neighboring parietal, opercular area OP4 (dysgranular) (Eickhoff et al., 2006c) showed a smaller size of pyramidal cells in the lower layer III and a lower cell density in layers III and V (Figure 6A). The main difference between area Op5 and the insular area Id4 (dysgranular) (Quabs et al., 2021a) was that layer III of Id4 was more sparsely populated with cells than layer III of Op5 (Figure 6B). The comparison of areas Op6 and premotor cortex revealed a weak laminar differentiation of area 6r1, i.e., all cortical layers from layer II to VI showed an approximately similar cell packing density (Amunts et al., 2010; Figure 6C). Like area 44, both areas Op6 and Op7 contained large pyramidal cells in layer III. However, they did not reach the size of pyramidal cells of area 44 (deep layer III and layer V) (Figures 6D, E). Compared with area Op7, area Op8 (Saal et al., 2021a) revealed a faint but recognizable layer IV in comparison with the prominent horizontal stripes of Op7 consisting of layers IIIc, IV, and Va. In adjacent layers, Op7 showed a lower cell density, and Op8 a higher cell density (especially in the lower layer III and in the upper layer V) (Figure 6F). The comparison of areas Op7 and Id6 (dysgranular) (Quabs et al., 2021b) revealed that pyramidal cells of layer IIIc had a higher dispersion, while the horizontal stripe in area Id6 appeared broader than in Op7 (Quabs et al., 2022; Figure 6G). In contrast to area Id7, Op7 showed a higher density of pyramidal cells in deep layer III and a higher packing of layer VI (Grodzinsky et al., 2020; Figure 6H).

### 3.2. Topography of areas Op5, Op6, and Op7

**FIGURE 7:**
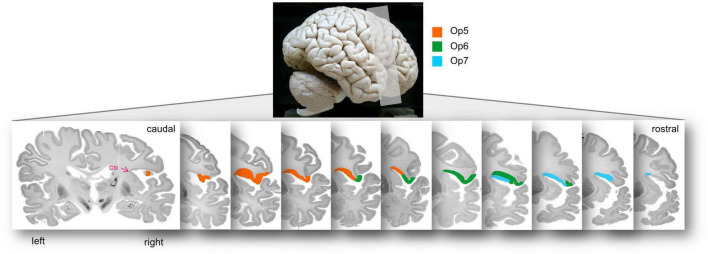
Localization of areas Op5, Op6, and Op7 in the right hemisphere of brain BC20 in serial coronal sections. The opercular areas Op5–Op7 approached to the insula up to the circular sulcus of the insula (CSI), which is highlighted with the pink arrowhead in the most posterior section. The upper lateral view of the brain illustrates the range of sectioning.

Areas Op5-Op7 occupied the posterior part of the FOp, and approached the insula, up to the circular sulcus of the insula (CSI). The borders between Op5 and Op6 as well as between Op6 and Op7 were always located lateral to the CSI, and never reached the medial wall (Figure 7 for the location of areas Op5–Op7 on consecutive sections).

Main parts of the three FOp-areas were located in the depths of the lateral fissure. In six of the 20 hemispheres, Op5 extended a few millimeters to the free brain surface (subcentral gyrus; two left hemispheres, four right hemispheres). In all hemispheres, Op5 reached the CSI and was located medially and anterior to area OP4. The border between Op5 and Op6 was in the ventral wall of the FOp.

In all 20 hemispheres, Op6 extended a few millimeters to the free brain surface (precentral gyrus/IFG). Op6 adjoined Op5 anteriorly and Op7 posteriorly. Lateral neighbors of Op6 were premotor area 6r1 and area 44 in caudo-rostral direction. Op7 reached the free brain surface in none of the hemispheres, but always was buried in the sulcus. Op7 was laterally adjacent to the insular cortex. Furthermore, Op7 was medially and posteriorly adjacent to area 44 and to the anterior part of the FOp (mainly Op8, more rarely Op9) (Saal et al., 2021a,b). Like the border between Op5 and Op6, the border between Op6 and Op7 was located in the depth of the fissure.

The medial neighbor of areas Op5, Op6, and Op7 was the anterior insula (Op5/Op6: Id4, Id6; Op7: Id6, Id7) (Grodzinsky et al., 2020; Quabs et al., 2021a,b). In some hemispheres, the posterior part of area Op5 was located medially to OP3 (Eickhoff et al., 2006c). Areas Op5-Op7 were mainly arranged in anterior-posterior direction. This localization did not differ between male and female brains.

### 3.3. Cytoarchitectonic 3D maps and intersubject variability

**FIGURE 8:**
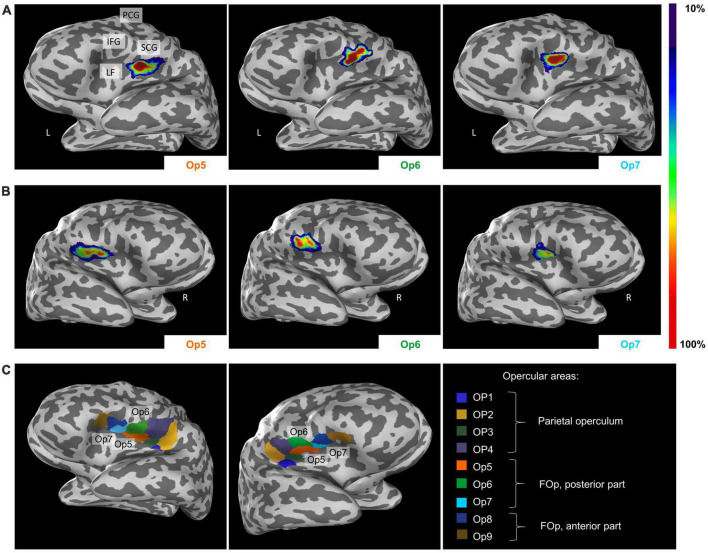
Lateral view from the left **(A)** and the right side **(B)** of the cytoarchitectonic probabilistic maps of the delineated areas Op5, Op6, and Op7 (MNI-Colin27 reference space, inflated view, Julich-Brain Atlas https://julich-brain-atlas.de/). The number of overlapping brains per voxel is color-coded. IFG, inferior frontal gyrus; LF, lateral fissure; PCG, precentral gyrus; SCG, subcentral gyrus. **(C)** Left-hemispheric and right-hemispheric maximum probability maps (MPMs) of areas Op5–Op7 in MNI-Colin27 reference space. The visualization also includes neighboring areas OP1–OP4 and Op8–Op9. Presentation of the MPMs on inflated brains to visualize also areas in sulci.

Intersubject variability in extent and location of areas Op5, Op6, and Op7 was quantified in the probability maps (Figure 8). The superimposition of all ten brains resulted in probabilistic maps, whereby the color coding from dark blue to red corresponds to an increasing overlap, or decreasing intersubject variability. Centers of gravity in 3D space for the left and right hemispheres are listed in Table 2; they did not differ between male and female brains.

**TABLE 2:** Coordinates of the centers of gravity for FOp areas Op5, Op6, and Op7 in MNI ICBM 152 space (upper table section) and anatomical MNI Colin27 space (lower table section) separated for the left and right hemisphere.

Center of gravity coordinates in MNI ICBM 152 space						
**Area**	**Left hemisphere**			**Right hemisphere**		
	**X** **Sagittal**	**Y** **Coronal**	**Z** **Horizontal**	**X** **Sagittal**	**Y** **Coronal**	**Z** **Horizontal**
Op5	−47	−5	13	45	−1	12
Op6	−52	5	6	52	8	5
Op7	−40	10	9	37	14	10
**Center of gravity coordinates in anatomical MNI Colin 27 space of continuous probability maps**						
**Area**	**Left hemisphere**			**Right hemisphere**		
	**X** **Sagittal**	**Y** **Coronal**	**Z** **Horizontal**	**X** **Sagittal**	**Y** **Coronal**	**Z** **Horizontal**
Op5	−47	−4	13	46	−2	12
Op6	−52	5	7	54	6	5
Op7	−39	10	11	39	12	10

The MPM reveals small parts of Op5 at the subcentral gyrus, while Op6 occupied parts of the precentral gyrus (Figure 8C). Op7 remained consistently within the lateral fissure. Cytoarchitectonic maps are shown also as surface representations in the reference brain Colin27 (Evans et al., 2012), to give a better impression of the extent of the areas in the depth of the brain. All data are part of the Julich-Brain Atlas (Amunts et al., 2020), and publicly available via the Human Brain Atlas of the EBRAINS research infrastructure of the Human Brain Project^3^.

### 3.4. Volumes of areas Op5, Op6, and Op7

**FIGURE 9:**
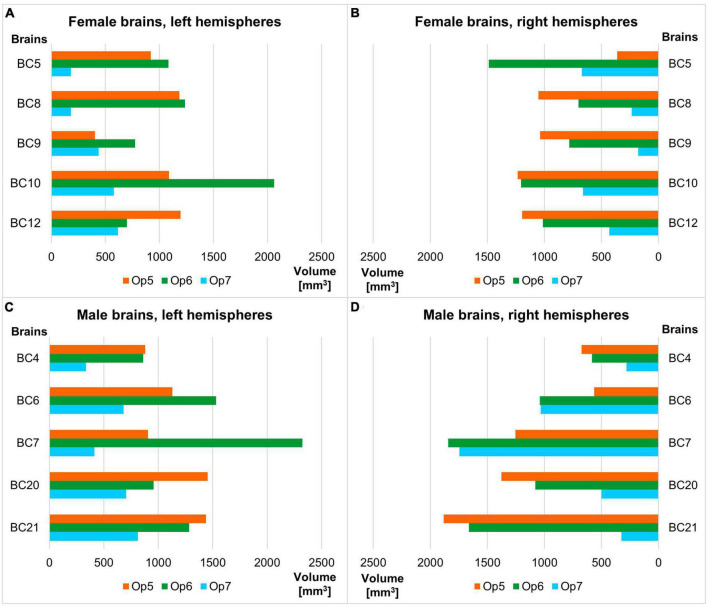
Volume of areas Op5–Op7 of the human FOp in each hemisphere of the ten postmortem brains sorted by female **(A,B)** and male brains **(C,D)**, each by left and right hemisphere. Analyses revealed no significant volume difference between sexes nor between hemispheres (all *p* > 0.05).

Areas Op5 and Op6 were about the same size, while area Op7 was the smallest area among the three. The intersubject variability of volume was most prominent for area Op6 and least prominent for area Op7 (see Table 3 for individual volumes per hemisphere). The shrinkage-corrected volumes did neither differ between hemispheres or sexes (all *p* > 0.05; see Table 4 and Figure 9).

**TABLE 3:** Size of Op5, Op6, and Op7 in both hemispheres.

Area	V_mean_ [mm^3^] ± SD [mm^3^]	
	**Left hemisphere**	**Right hemisphere**
Op5	1,059.98 ± 305.97	1,142.91 ± 453.18
Op6	1,281.77 ± 545.07	1,139.33 ± 415.07
Op7	525.32 ± 190.34	509.79 ± 313.81

Mean values (V_means_) of the corrected volumes with associated standard deviations (SD) for Op5, Op6, and Op7, separated for the left and right hemispheres. Histologic volumes were corrected using the individual shrinkage factor of each individual brain.

**TABLE 4:** Volume of areas Op5–Op7 of the human FOp in each hemisphere of the 10 postmortem brains.

Brain	Areal volume [mm^3^]						Sum
	**Op5 left**	**Op5 right**	**Op6 left**	**Op6 right**	**Op7 left**	**Op7 right**	
BC4	880.64	673.62	862.81	582.65	337.48	280.21	3,617.41
BC5	918.83	362.48	1,084.69	1,488.09	181.44	671.16	4,706.69
BC6	1,129.95	1,032.09	1,530.89	1,039.16	681.33	564.37	5,977.80
BC7	905.44	1,745.84	2,324.69	1,842.41	413.13	1,254.46	8,485.98
BC8	1,185.68	1,052.69	1,236,78	701,92	487.63	233.74	4898.44
BC9	404.81	1,038.41	774.47	781.92	438.48	177.97	3,616.06
BC10	1,087.60	1,234.67	2,061.01	1,204.37	578.37	661.69	6,827,71
BC12	1,195.72	1,029.52	700.63	1,013.90	616.89	430.58	4,987.24
BC20	1,453.47	1,376.31	957.07	1,078.14	704.52	499.13	6,068.64
BC21	1,437.65	1,883.46	1,284.70	1,660.74	813.97	324.63	7,405.14
Mean	1,059.98	1,142.91	1,281.77	1,139.33	525.32	509.79	5,659.11
SD	305.971	453.18	545.07	415.07	190.34	313.81	2,223.43

### 3.5. Functional profiles of Op5, Op6, and Op7

Based on the BrainMap database, area-specific functions of Op5, Op6, and Op7 were analyzed. Related Sleuth search results from the BrainMap database can be found in Supplementary Table 2. All results of the behavioral domain category as well as of the paradigm class category can be found in Table 5.

**TABLE 5:** Behavioral domains and paradigm classes of Op5, Op6, and Op7 left and right (*p* < 0.05, FDR corrected) represented by colored boxes per hemisphere.

Behavioral domain	Op5		Op6		Op7	
	**LH**	**RH**	**LH**	**RH**	**LH**	**RH**
Cognition.Music						
Interoception sexuality						
Perception somesthesis.Pain						
**Paradigm class**	**Op5**		**Op6**		**Op7**	
	**LH**	**RH**	**LH**	**RH**	**LH**	**RH**
Music comprehension						
Flexion/Extension						
Chewing/Swallowing						
Sexual arousal/Gratification						
Isometric force						
Pain monitor/Discrimination						

LH, left hemisphere; RH, right hemisphere.

The following functional profiles were found: Op6 left was involved in music cognition, whereas Op5 right was active in chewing/swallowing and sexual processing. Both areas together showed an activation in isometric force of muscles. Op6 right showed an activation in flexion/extension.

### 3.6. Functional and structural connectivity on areas Op5, Op6, and Op7

**FIGURE 10:**
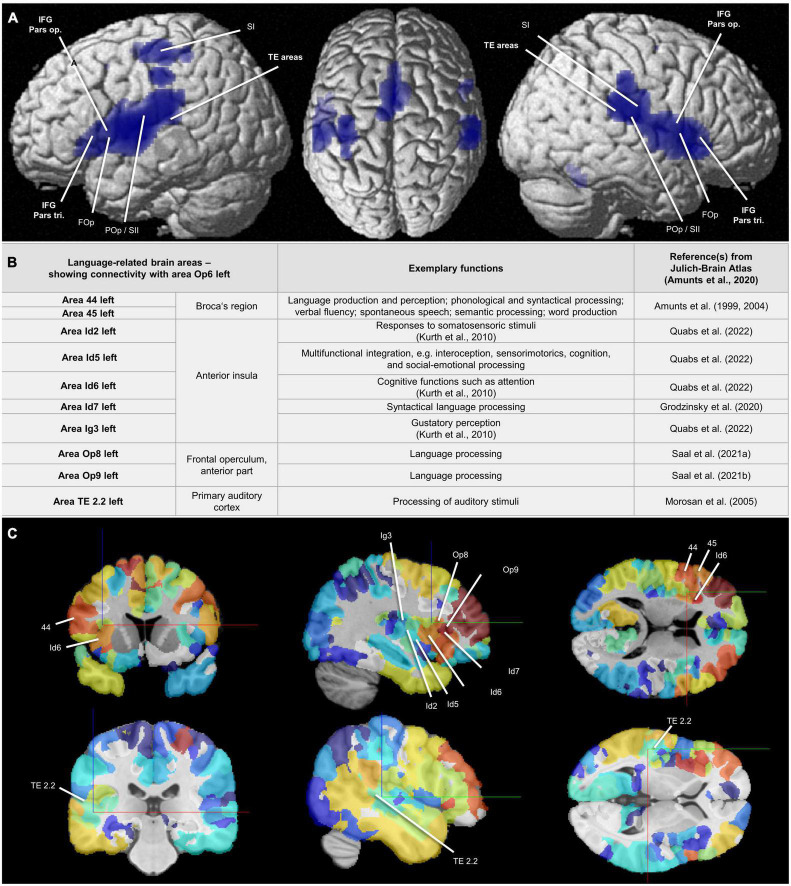
**(A)** Areas Op5–Op7: Results of the conjunction analysis displayed on the cortical surface (MNI ICBM 152 reference brain) (Nichols et al., 2005) proving combined co-activation of the areas Op5-Op7 calculated by meta-analytic connectivity modeling (MACM) (Eickhoff et al., 2011). The analysis revealed that areas Op5-Op7 showed co-activational connectivity with area 44 (Broca’s region), with the FOp, and with the TE areas. Also with the POp and and with parts of the somatosensory cortex, all FOp areas showed co-activational connectivity. FOp = frontal operculum; IFG Pars op. = inferior frontal gyrus, opercular part; IFG Pars tri. = inferior frontal gyrus, triangular part; POp/SII = parietal operculum/secondary somatosensory cortex; SI = primary somatosensory cortex; TE areas = auditory cortex in the temporal lobe. **(B)** Area Op6 left: Language-related brain areas that showed connectivity with area Op6 left in the present study and thus were connected with the FOp in a network. For full connectivity results, see Supplementary Tables 3, 4. Additional reference: Kurth et al. (2010)—for citation, see Supplementary material. **(C)** Structural connectivity of area Op6 left: Frontal, sagittal, and coronal sections in the MNI ICBM 152 reference space (two different cutting heights). Marked are areas associated with selected functions including language, which show a structural connectivity to Op6 left. Level of structural connectivity to Op6 left from the 1000BRAINS cohort (Caspers et al., 2014) is indicated by coloring of areas red (high structural connectivity) to blue (low structural connectivity). Physical coordinates (cursor) for upper **(C)**: –36.095 mm/10.771 mm/12.499 mm; Physical coordinates (cursor) for lower **(C)**: –51.450 mm/–29.050 mm/2.100 mm (https://atlases.ebrains.eu/viewer).

The conjunction analysis of MACM revealed that areas Op5–Op7 showed co-activational connectivity with area 44 (Broca’s region), with the parietal operculum (POp), with areas of the temporal lobe as well as with parts of the somatosensory cortex (Figure 10A). The contrast analysis of MACM showed differences in co-activation by comparing two of the new opercular maps. An overview of all MACM results is summarized in Supplementary Table 3. Areas of co-activational clustering were connected with the cytoarchitectonically defined areas of the Julich-Brain Atlas 2.9 (Amunts et al., 2020; Supplementary Tables 3–4).

Op5 left showed co-activations with area 1 (Geyer et al., 1999, 2000), area 2 (Grefkes et al., 2001), area 3b (Geyer et al., 1999, 2000), area Id4 (Quabs et al., 2022), and area OP3 (Eickhoff et al., 2006a,c) in the left hemisphere as well as with area Id6 (Quabs et al., 2022) in the right hemisphere. Op5 right showed co-activations with areas 3a (Geyer et al., 1999, 2000), 3b, 4a (Geyer et al., 1996), 4p (Geyer et al., 1996), and OP1 (Eickhoff et al., 2006a,c) in the left hemisphere and with areas OP3 and OP4 (Eickhoff et al., 2006a,c) in the right hemisphere. Additional co-activations were detected for Op5 right with 6ma (Ruan et al., 2018), 6mp (Ruan et al., 2018), the medial geniculate body (Kiwitz et al., 2022), and Id4 in both hemispheres.

Op6 left showed co-activations with area 44 (Amunts et al., 1999, 2004), area Id6, area FG1 (Caspers et al., 2013), and area FG2 (Caspers et al., 2013), in the left hemisphere as well as with areas hIP1 (Choi et al., 2006), hIP3 (Scheperjans et al., 2008a,b), and PFm (inferior parietal lobule, IPL) (Caspers et al., 2006, 2008) in the right hemisphere. Further co-activations existed with areas 6ma and FG4 (Lorenz et al., 2017) in both hemispheres. For Op6 right, co-activations with areas 6mp, OP1, OP4, PF (IPL) (Caspers et al., 2006, 2008) and PFcm (IPL) (Caspers et al., 2006, 2008) right as well as with bilateral area 6ma could be observed.

Op7 left showed co-activations with areas 33 (Palomero-Gallagher et al., 2015) and Id7 (Grodzinsky et al., 2020) in the right hemisphere as well as with area Id6 in both hemispheres. Further co-activations could be observed for Op7 right with OP1 right as well as with bilateral areas Id6 and Id7.

In comparison to the MACM results, the structural connectivity analysis based on the 1000BRAINS study (results in alphabetical order) revealed structural connectivity of Op5 left with Id4, Id6, Ig3 (Quabs et al., 2022), OP1, OP3, and OP4 in the left hemisphere. For Op5 right, there was structural connectivity with areas 3b, 4a, 6d1 (Sigl et al., 2021a), 6d2 (Sigl et al., 2021b), 6ma, Id4, Id5 (Quabs et al., 2022), Id6, Id7, OP1-OP4, PF, PFcm, PFm, PFop (IPL) (Caspers et al., 2006, 2008), and PFt (IPL) (Caspers et al., 2006, 2008) in the right hemisphere. For Op6 left, structural connectivity could be observed with areas 1, 4a, 44, 45 (Amunts et al., 1999, 2004), Id2 (Quabs et al., 2022), Id5-Id7, Ig3, Op8 (Saal et al., 2021a), Op9 (Saal et al., 2021b), PF, PFcm, PFm, PFop, PFt, and TE 2.2

(Morosan et al., 2005) in the left hemisphere as well as with 6d1 and 6d2 in both hemispheres. For Op6 right, there was structural connectivity with areas 6d1, 6d2, 6d3 (Sigl et al., 2021c), 6ma, Id4, OP4, PFm, PFop, PFt, and PGa (Caspers et al., 2006, 2008) right. For Op7 left remained structural connectivity with Id6 left. For Op7 right, there was structural connectivity with Id5 and Id6 right. Selected functional and structural connectivity results, specifically for area Op6 left, can be found in Figures 10B, C.

## 4. Discussion

The present study revealed a new microstructural parcellation of the posterior FOp in the human brain based on cytoarchitectonic differences and introduced 3D maps of the three new areas Op5, Op6, and Op7. Cytoarchitectonic borders were identified using image analysis and statistical criteria, to make them reproducible. The new areas were cytoarchitectonically distinct from the adjacent insula and other surrounding areas. The varying location and extent of the FOp-areas were reflected in cytoarchitectonic probabilistic maps in the MNI-Colin27 and the MNI ICBM 152 space, which enable analyses and comparisons with *in vivo* neuroimaging data serving as anatomical reference. No significant differences in the volumes, localization of areas Op5-Op7 in space, or with respect to their relationship to sulci and gyri were found between male and female brains.

The present study confirmed our hypothesis that the FOp is composed of several cytoarchitectonic areas that are distinct in microstructure and connectivity (Anwander et al., 2007; Amunts et al., 2010; Friederici, 2017). By evaluating MACM and structural connectivity results and by comparing co-activations and functional specifics of the co-activated areas, we identified distinct functions, with Op6 left being involved in music cognition and wider music/language-relevant networks. Combined with Op5 right, Op6 left was also active in a task of isometric force of muscles. Op5 right showed involvement in chewing/swallowing and sexual processing, while Op6 right was active in flexion/extension.

### 4.1. Comparison of new maps of Op5-Op7 with other maps of the FOp

The present results supplement earlier findings of our group based on receptorarchitectonic analyses (Amunts et al., 2010), and go beyond the parcellation of the FOp in historical maps, proposed, for example, by Brodmann (1909) and von Economo and Koskinas (1925). Brodmann described BA 43 and BA 6 near the central sulcus as two homogeneous areas, but his drawing only shows the brain surface. The new Op5 of the present study most likely corresponds to the rostral part of BA 43 (Brodmann, 1909). Areas Op6 and Op7 seemed to correspond to the caudal part of BA 6, partially adjacent to BA 43 (Brodmann, 1909). The putative anatomical correlates for Op5-Op7 in the maps of von Economo and Koskinas (1925) are most probably areas PF*D*, FB_op_, FC*D*_op_, and FD_op_. However, the pattern of segregation is not the same. While Op5 may mainly be assigned to the rostral part of PF*D* and to the caudal part of FB_op_, areas Op6 and Op7 seem to correspond to the rostral part of FB_op_, to area FC*D*_op_, and to the most caudal part of FD_op_ (von Economo and Koskinas, 1925). Clearly, a more thorough comparison with previous maps would require aligning them to the same reference space, which is not an option.

More recent maps of the human prefrontal cortex of Petrides and Pandya (1994) showed an extended area 6 in the peri-Sylvian region, adjacent to the central sulcus. Area 6 of Petrides and Pandya (1994) consisted of the areas 6VR and 6VC (in anterior-posterior direction). Area PrCO of Öngür et al. (2003) most probably corresponds to areas Op6 and Op7, based on their description of an area anterior to the central sulcus within the lateral fissure.

In accordance to previous findings (e.g., Keller et al., 2009), no anatomical asymmetry or lateralization of the FOp in favor of the left hemisphere could be found for Op5–Op7. This is different from the finding of an anatomical asymmetry of area 44. In area 44, a left-hemispheric asymmetry in volume could be observed providing a putative structural correlate for lateralization of speech (Amunts et al., 1999).

### 4.2. Functional decoding of areas Op5, Op6, and Op7

The MACM analyses revealed a contribution of left Op6 to music cognition. This is in correspondence with earlier studies showing an association of the entire FOp with rhythm perception (Limb et al., 2006), musical priming (intonation judgments) (Tillmann et al., 2003), and song complexity (Brown et al., 2004). The present analysis also showed a co-activation of Op6 left with area 44 in the left hemisphere. 1000BRAINS data confirmed this by showing structural connectivity of Op6 left with left area 44 and additionally, with left area 45. As for the FOp, there was evidence for Broca’s region being involved in music processing (Levitin and Menon, 2003), which fits in that music and language are both based on syntactic rules that are complex, hierarchically-structured, and based on implicitly existing structural norms (Kunert et al., 2015).

For right area Op5, the MACM analysis indicated a connection to interoception of sexuality. A perfusion fMRI investigation about male sexual activity showed that the whole FOp is a brain region correlated with measures of sexual behavior (Georgiadis et al., 2010). The FOp was also mentioned as neuroanatomical correlate of penile erection when viewing photographic stimuli (Moulier et al., 2006). MACM revealed co-activations between area Op5 right and areas 3a, 3b, 4a, 4p, the medial geniculate body, Id4, OP1, and OP3, which corresponds largely (except the precentral gyrus) to the areas that have been associated with sexual behavioral measures (Georgiadis et al., 2010). Blood oxygen level-dependent effects described by Moulier et al. (2006), however, confirmed also the precentral co-activations for the FOp shown by MACM, and were also reflected in the structural connectivity data considered. At the same time, the underlying cytoarchitecture did not differ between male and female brains. A larger sample size might be necessary to reveal such differences, which would go beyond the scope of the present study.

For right area Op5, functional decoding with MACM revealed an association with chewing/swallowing. This is in agreement to a study of Lowell et al. (2009), where an involvement of the FOp in overt swallowing was revealed. The POp, the supplementary motor area, and several parts of the thalamus also showed activation for the swallowing task in the study, which fits to the co-activations of the right Op5 with the medial geniculate body as well as with areas OP1, OP3, and OP4 in MACM. Further co-activations for right Op5 could be observed with motor areas 4a, 4p, 6ma, 6mp, and Id4. The relationship to motor areas was further confirmed by the analysis of structural connectivity, which was demonstrated for area OP2, motor areas 6d1, 6d2, and parts of the inferior parietal lobule (PF, PFcm, PFm, PFop, and PFt).

For Op6 left as well as for Op5 right, the MACM analysis revealed a functional involvement in studies of isometric force of muscles. A study by Ward and Frackowiak (2003) about age-dependent changes in neural correlates during an isometric hand grip task showed age-varying activations in the FOp. For Op6 left, the MACM analysis showed also correlations with other areas involved in the “grasping circuit” as described by Ward and Frackowiak (2003), including area 44 left as well as areas hIP1 and hIP3 of the intraparietal sulcus. Structural connectivity data affirmed connections of left Op6 to left area 44. Furthermore, it showed structural connections to areas 1 and 4a in combination with parts of area 6 and the IPL, being relevant for motor functions (Caspers et al., 2006, 2008).

For right area Op6, the MACM analysis indicated activation during flexion/extension. In a study in which older participants had to perform a bimanual flexion/extension task, the FOp was one of the activated areas (Goble et al., 2010). The fact that MACM showed co-activation for Op6 right with areas 6ma, 6mp, OP1, and OP4 fits to activations of motor area and SII region that were additionally mentioned by Goble et al. (2010). In addition to motor area 6 and the POp, by including structural connectivity, connections to the area Id4 and IPL areas were found.

### 4.3. Specificity of areas Op5-Op7 in music/language versus other functions

The analyses with BrainMap suggested that left area Op6 is involved in music-related functions, which has also been reported for Broca’s region (Chiang et al., 2018). Op6, in particular, shows cytoarchitectonic similarities with neighboring area 44: Both are dysgranular, with large pyramidal cells in layers III and V (although a bit smaller in Op6 than in BA 44). Furthermore, both areas showed functional co-activations with each other according to the present MACM. The analysis of the structural connectivity of left Op6 based on the 1000BRAINS study indicated that it is connected with left areas 44 and 45. Similarities in music perception and syntactic processing of language have been discussed (Kunert et al., 2015), and one might argue that Op6 is also involved in both. Similar to connections of Broca’s region with the superior temporal gyrus [(Catani et al., 2005; Friederici, 2009, 2011, 2012; Fernández-Miranda et al., 2015), for reviews see Bernal and Ardila (2009) and Bernard et al. (2019)], the connection of Op6 left with left area TE 2.2 of the extended Wernicke’s region could indicate a supporting role of Op6 left in the language network.

Although both FOp and Broca’s region support music and language functions (e.g., Golestani and Zatorre, 2004; Friederici et al., 2006a; Grodzinsky and Friederici, 2006; Zysset et al., 2007; Ventura-Campos et al., 2013; Chai et al., 2016), left Op6 is also functionally distinct from Broca’s region. In terms of syntactic processing, for example, studies by Friederici and colleagues showed that the left FOp is involved in building local phrase structures (noun phrases, verb phrases as well as prepositional phrases), while Broca’s region has been shown to support the computation of dependency relation between constituents of a sentence and to play a crucial role in the processing of syntactically complex sentence hierarchies (Grodzinsky, 2000; Friederici et al., 2003, 2006a; Friederici, 2006; Grodzinsky and Friederici, 2006).

Previously reported differences on functional and connectivity-based heterogeneity between the genders regarding phonology (Shaywitz et al., 1995) could not be verified on a microstructural level, neither by the hierarchical cluster analysis nor by the volume analysis. Again, the relatively small sample size in this mapping study represents a limitation for such type of analysis.

Interestingly, the study confirmed an involvement of the identified opercular areas in functional networks different from language and music processing. Area Op5 right was associated with sexual processing based on functional decoding and multiple connections have been found with the POp. This finding may indicate a role of Op5 right in the cortical somatosensory networks. Eickhoff et al. (2006b) discussed that the parietal opercular cortex receives various sensory information and integrates them into an interoceptive perception of the body’s condition (Eickhoff et al., 2006b). In addition, areas Op5 and Op6 seem to be part of motor control networks. Functional integration of chewing/swallowing, isometric force of muscles, and flexion/extension was reflected by structural connections of area Op5 right to areas 6d1, 6d2, and 6ma as well as of area Op6 left to areas 6d1 and 6d2. In terms of swallowing, also connections to the POp (areas OP1 OP3, and OP4) were relevant (Lowell et al., 2009). Area Op5 is a (rostral) direct neighbor of the POp, which is associated with somesthesis, while Op6 is a direct (ventral) neighbor of area 44 linked to music and language and area 6 linked to motor functions. Thus, it could be hypothesized that the posterior part of the FOp could also serve a kind of transition zone between these areas.

### 4.4. Data availability

The new maps of areas Op5, Op6, and Op7 are openly available in different reference spaces (MNI-Colin27 and MNI ICBM 152 reference spaces) and as surface representation. The maps are part of the Julich-Brain Atlas^4^ and can be explored using the 3D atlas viewer “siibra-explorer” and other tools of the siibra toolsuite of the EBRAINS research infrastructure (see footnote 2) of the Human Brain Project. They can be downloaded as part of the Julich-Brain 2.9 cytoarchitectonic maps,^5^ or as individual datasets [Op5,^6^ (Unger et al., 2021a); Op6,^7^ (Unger et al., 2021b); Op7,^8^ (Unger et al., 2021c)]. Areas Op5-Op7 were also mapped in the BigBrain (brain BC20) (Amunts et al., 2013), and maps are also available in the BigBrain space. For each map, region-specific datasets are linked via the EBRAINS Knowledge Graph^9^ and the siibra toolsuite (siibra-explorer hosted at https://atlases.ebrains.eu/viewer; siibra-python client: https://siibra-python.readthedocs.io).

### 4.5. Conclusion

The present study provided a comprehensive cytoarchitectonic description and maps of the posterior part of the FOp consisting of areas Op5, Op6, and Op7. The areas not only proved to be different in terms of cytoarchitecture, but also showed functional differences, which corresponds to prior receptor studies. Op6 left was shown to play a role in music processing, and a role in the broader language networks could be assumed. While sexual sensations could be assigned to Op5 right, motor connections could be found for Op5 and Op6. Thus, the present study was able to characterize the posterior part of the FOp more precisely, both structurally and functionally. The openly available maps will serve as a tool to decode the function of this region in more detail.

## Data availability statement

The datasets presented in this study can be found in online repositories. The names of the repository/repositories and accession number(s) can be found in the article/Supplementary material.

## Ethics statement

The studies involving human participants were reviewed and approved by the Ethics Committee of the Medical Faculty of the Heinrich Heine University Düsseldorf (permission #4863). Written informed consent for participation was not required for this study in accordance with the national legislation and the institutional requirements.

## Author contributions

NU performed and calculated cytoarchitectonic characterization, statistical border detection of the human FOp areas, and statistics with support by KA, SC, and SB. MH contributed to cytoarchitectonic mapping. KA designed the study and supervised all aspects of the study. NU prepared hierarchical cluster analysis and SB computed it. HM computed the volumetric analysis and NU and SB made the final analyses. HM calculated the probabilistic maps and MPMs. NU estimated the functional characterization and wrote the first draft of the manuscript. SBE, JAC, and TD conducted the MACM analysis and subsequently interpreted by NU. All authors performed the discussion of results, writing, and editing of the manuscript.

## Footnotes

1
http://brainmap.org

2
https://atlases.ebrains.eu/viewer

3
https://www.humanbrainproject.eu/en/explore-the-brain

4
https://julich-brain-atlas.de/

5
https://doi.org/10.25493/VSMK-H94

6
https://doi.org/10.25493/KN1A-YX4

7
https://doi.org/10.25493/RQKR-WE4

8
https://doi.org/10.25493/W2D1-DJF

9
https://kg.ebrains.eu/search

## References

[B1] Amunts K. Zilles K. (2012). Architecture and organizational principles of Broca’s region. *Trends Cogn. Sci.* 16 418–426. 10.1016/j.tics.2012.06.005 22763211

[B2] Amunts K. Zilles K. (2015). Architectonic mapping of the human brain beyond Brodmann. *Neuron* 88 1086–1107. 10.1016/j.neuron.2015.12.001 26687219

[B3] Amunts K. Lenzen M. Friederici A. D. Schleicher A. Morosan P. Palomero-Gallagher N. (2010). Broca’s region: Novel organizational principles and multiple receptor mapping. *PLoS Biol.* 8:e1000489. 10.1371/journal.pbio.1000489 20877713 PMC2943440

[B4] Amunts K. Lepage C. Borgeat L. Mohlberg H. Dickscheid T. Rousseau M.-É. (2013). BigBrain: An ultrahigh-resolution 3D human brain model. *Science* 340 1472–1475. 10.1126/science.1235381 23788795

[B5] Amunts K. Mohlberg H. Bludau S. Zilles K. (2020). Julich-Brain: A 3D probabilistic atlas of the human brain’s cytoarchitecture. *Science* 369 988–992. 10.1126/science.abb4588 32732281

[B6] Amunts K. Schleicher A. Zilles K. (2004). Outstanding language competence and cytoarchitecture in Broca’s speech region. *Brain Lang.* 89 346–353. 10.1016/S0093-934X(03)00360-2 15068917

[B7] Amunts K. Schleicher A. Bürgel U. Mohlberg H. Uylings H. B. M. Zilles K. (1999). Broca’s region revisited: Cytoarchitecture and intersubject variability. *J. Comp. Neurol.* 412 319–341. 10.1002/(SICI)1096-9861(19990920)412:2<319::AID-CNE10>3.0.CO;2-7 10441759

[B8] Anwander A. Tittgemeyer M. Von Cramon D. Y. Friederici A. D. Knösche T. R. (2007). Connectivity-based parcellation of Broca’s area. *Cereb. Cortex* 17 816–825. 10.1093/cercor/bhk034 16707738

[B9] Bernal B. Ardila A. (2009). The role of the arcuate fasciculus in conduction aphasia. *Brain* 132 2309–2316. 10.1093/brain/awp206 19690094

[B10] Bernard F. Zemmoura I. Ter Minassian A. Lemée J.-M. Menei P. (2019). Anatomical variability of the arcuate fasciculus: A systematical review. *Surg. Radiol. Anat.* 41 889–900. 10.1007/s00276-019-02244-5 31028450

[B11] Binder J. R. (2017). Current controversies on Wernicke’s area and its role in language. *Curr. Neurol. Neurosci. Rep.* 17:58. 10.1007/s11910-017-0764-8 28656532

[B12] Bludau S. Eickhoff S. B. Mohlberg H. Caspers S. Laird A. R. Fox P. T. (2014). Cytoarchitecture, probability maps and functions of the human frontal pole. *Neuroimage* 93(Pt 2) 260–275. 10.1016/j.neuroimage.2013.05.052 23702412 PMC5325035

[B13] Bohland J. W. Guenther F. H. (2006). An fMRI investigation of syllable sequence production. *Neuroimage* 32 821–841. 10.1016/j.neuroimage.2006.04.173 16730195

[B14] Brodmann K. (1909). *Vergleichende Lokalisationslehre der Großhirnrinde.* Leipzig: Barth.

[B15] Brown S. Martinez M. J. Hodges D. A. Fox P. T. Parsons L. M. (2004). The song system of the human brain. *Cogn. Brain Res.* 20 363–375. 10.1016/j.cogbrainres.2004.03.016 15268914

[B16] Caspers J. Zilles K. Eickhoff S. B. Schleicher A. Mohlberg H. Amunts K. (2013). Cytoarchitectonical analysis and probabilistic mapping of two extrastriate areas of the human posterior fusiform gyrus. *Brain Struct. Funct.* 218 511–526. 10.1007/s00429-012-0411-8 22488096 PMC3580145

[B17] Caspers S. Schreiber J. (2021). *HBP data descriptor: 1000BRAINS study, connectivity data (v1.1).* Available online at: https://search.kg.ebrains.eu/instances/83407c06-b494-4307-861e-d06a5aecdf8a (accessed July 10, 2022).

[B18] Caspers S. Eickhoff S. B. Geyer S. Scheperjans F. Mohlberg H. Zilles K. (2008). The human inferior parietal lobule in stereotaxic space. *Brain Struct. Funct.* 212 481–495. 10.1007/s00429-008-0195-z 18651173

[B19] Caspers S. Geyer S. Schleicher A. Mohlberg H. Amunts K. Zilles K. (2006). The human inferior parietal cortex: Cytoarchitectonic parcellation and interindividual variability. *Neuroimage* 33 430–448. 10.1016/j.neuroimage.2006.06.054 16949304

[B20] Caspers S. Moebus S. Lux S. Pundt N. Schütz H. Mühleisen T. W. (2014). Studying variability in human brain aging in a population-based German cohort-rationale and design of 1000BRAINS. *Front. Aging Neurosci.* 6:149. 10.3389/fnagi.2014.00149 25071558 PMC4094912

[B21] Catani M. Jones D. K. Ffytche D. H. (2005). Perisylvian language networks of the human brain. *Ann. Neurol.* 57 8–16. 10.1002/ana.20319 15597383

[B22] Chai X. J. Berken J. A. Barbeau E. B. Soles J. Callahan M. Chen J.-K. (2016). Intrinsic functional connectivity in the adult brain and success in second-language learning. *J. Neurosci.* 36 755–761. 10.1523/JNEUROSCI.2234-15.2016 26791206 PMC6602001

[B23] Chiang J. N. Rosenberg M. H. Bufford C. A. Stephens D. Lysy A. Monti M. M. (2018). The language of music: Common neural codes for structured sequences in music and natural language. *Brain Lang.* 185 30–37. 10.1016/j.bandl.2018.07.003 30086421

[B24] Chikazoe J. Lee D. H. Kriegeskorte N. Anderson A. K. (2019). Distinct representations of basic taste qualities in human gustatory cortex. *Nat. Commun.* 10:1048. 10.1038/s41467-019-08857-z 30837463 PMC6401093

[B25] Choi H.-J. Zilles K. Mohlberg H. Schleicher A. Fink G. R. Armstrong E. (2006). Cytoarchitectonic identification and probabilistic mapping of two distinct areas within the anterior ventral bank of the human intraparietal sulcus. *J. Comp. Neurol.* 495 53–69. 10.1002/cne.20849 16432904 PMC3429851

[B26] DeWitt I. Rauschecker J. P. (2013). Wernicke’s area revisited: Parallel streams and word processing. *Brain Lang.* 127 181–191. 10.1016/j.bandl.2013.09.014 24404576 PMC4098851

[B27] Eickhoff S. B. Lotze M. Wietek B. Amunts K. Enck P. Zilles K. (2006b). Segregation of visceral and somatosensory afferents: An fMRI and cytoarchitectonic mapping study. *Neuroimage* 31 1004–1014. 10.1016/j.neuroimage.2006.01.023 16529950

[B28] Eickhoff S. B. Schleicher A. Zilles K. Amunts K. (2006c). The human parietal operculum. I. Cytoarchitectonic mapping of subdivisions. *Cereb. Cortex* 16 254–267. 10.1093/cercor/bhi105 15888607

[B29] Eickhoff S. B. Amunts K. Mohlberg H. Zilles K. (2006a). The human parietal operculum. II. Stereotaxic maps and correlation with functional imaging results. *Cereb. Cortex* 16 268–279. 10.1093/cercor/bhi106 15888606

[B30] Eickhoff S. B. Bzdok D. Laird A. R. Roski C. Caspers S. Zilles K. (2011). Co-activation patterns distinguish cortical modules, their connectivity and functional differentiation. *Neuroimage* 57 938–949. 10.1016/j.neuroimage.2011.05.021 21609770 PMC3129435

[B31] Eickhoff S. B. Nichols T. E. Laird A. R. Hoffstaedter F. Amunts K. Fox P. T. (2016). Behavior, sensitivity, and power of activation likelihood estimation characterized by massive empirical simulation. *Neuroimage* 137 70–85. 10.1016/j.neuroimage.2016.04.072 27179606 PMC4981641

[B32] Eickhoff S. B. Paus T. Caspers S. Grosbras M.-H. Evans A. C. Zilles K. (2007). Assignment of functional activations to probabilistic cytoarchitectonic areas revisited. *Neuroimage* 36 511–521. 10.1016/j.neuroimage.2007.03.060 17499520

[B33] Eickhoff S. B. Stephan K. E. Mohlberg H. Grefkes C. Fink G. R. Amunts K. (2005). A new SPM toolbox for combining probabilistic cytoarchitectonic maps and functional imaging data. *Neuroimage* 25 1325–1335. 10.1016/j.neuroimage.2004.12.034 15850749

[B34] Evans A. C. Janke A. L. Collins D. L. Baillet S. (2012). Brain templates and atlases. *Neuroimage* 62 911–922. 10.1016/j.neuroimage.2012.01.024 22248580

[B35] Fedorenko E. Blank I. A. (2020). Broca’s area is not a natural kind. *Trends Cogn. Sci.* 24 270–284. 10.1016/j.tics.2020.01.001 32160565 PMC7211504

[B36] Fernández-Miranda J. C. Wang Y. Pathak S. Stefaneau L. Verstynen T. Yeh F.-C. (2015). Asymmetry, connectivity, and segmentation of the arcuate fascicle in the human brain. *Brain Struct. Funct.* 220 1665–1680. 10.1007/s00429-014-0751-7 24633827

[B37] Fiez J. A. Tranel D. Seager-Frerichs D. Damasio H. (2006). Specific reading and phonological processing deficits are associated with damage to the left frontal operculum. *Cortex* 42 624–643. 10.1016/s0010-9452(08)70399-x 16881271

[B38] Fox P. T. Lancaster J. L. (2002). Mapping context and content: The BrainMap model. *Nat. Rev. Neurosci.* 3 319–321. 10.1038/nrn789 11967563

[B39] Friederici A. D. (2006). Broca’s area and the ventral premotor cortex in language: Functional differentiation and specificity. *Cortex* 42 472–475. 10.1016/s0010-9452(08)70380-0 16881252

[B40] Friederici A. D. (2009). Pathways to language: Fiber tracts in the human brain. *Trends Cogn. Sci.* 13 175–181. 10.1016/j.tics.2009.01.001 19223226

[B41] Friederici A. D. (2011). The brain basis of language processing: From structure to function. *Physiol. Rev.* 91 1357–1392. 10.1152/physrev.00006.2011 22013214

[B42] Friederici A. D. (2012). Language development and the ontogeny of the dorsal pathway. *Front. Evol. Neurosci.* 4:3. 10.3389/fnevo.2012.00003 22347185 PMC3272640

[B43] Friederici A. D. (2017). *Language in our brain: The origins of a uniquely human capacity.* Cambridge, MA: MIT Press.

[B44] Friederici A. D. Bahlmann J. Heim S. Schubotz R. I. Anwander A. (2006a). The brain differentiates human and non-human grammars: Functional localization and structural connectivity. *Proc. Natl. Acad. Sci. U.S.A.* 103 2458–2463. 10.1073/pnas.0509389103 16461904 PMC1413709

[B45] Friederici A. D. Fiebach C. J. Schlesewsky M. Bornkessel I. D. Von Cramon D. Y. (2006b). Processing linguistic complexity and grammaticality in the left frontal cortex. *Cereb. Cortex* 16 1709–1717. 10.1093/cercor/bhj106 16400163

[B46] Friederici A. D. Opitz B. Von Cramon D. Y. (2000). Segregating semantic and syntactic aspects of processing in the human brain: An fMRI investigation of different word types. *Cereb. Cortex* 10 698–705. 10.1093/cercor/10.7.698 10906316

[B47] Friederici A. D. Rüschemeyer S.-A. Hahne A. Fiebach C. J. (2003). The role of left inferior frontal and superior temporal cortex in sentence comprehension: Localizing syntactic and semantic processes. *Cereb. Cortex* 13 170–177. 10.1093/cercor/13.2.170 12507948

[B48] Georgiadis J. R. Farrell M. J. Boessen R. Denton D. A. Gavrilescu M. Kortekaas R. (2010). Dynamic subcortical blood flow during male sexual activity with ecological validity: A perfusion fMRI study. *Neuroimage* 50 208–216. 10.1016/j.neuroimage.2009.12.034 20006720

[B49] Geyer S. Ledberg A. Schleicher A. Kinomura S. Schormann T. Bürgel U. (1996). Two different areas within the primary motor cortex of man. *Nature* 382, 805–807. 10.1038/382805a0 8752272

[B50] Geyer S. Schleicher A. Zilles K. (1999). Areas 3a, 3b, and 1 of human primary somatosensory cortex: 1. Microstructural organization and interindividual variability. *Neuroimage* 10 63–83. 10.1006/nimg.1999.0440 10385582

[B51] Geyer S. Schormann T. Mohlberg H. Zilles K. (2000). Areas 3a, 3b, and 1 of human primary somatosensory cortex: 2. Spatial normalization to standard anatomical space. *Neuroimage* 11 684–696. 10.1006/nimg.2000.0548 10860796

[B52] Goble D. J. Coxon J. P. Van Impe A. De Vos J. Wenderoth N. Swinnen S. P. (2010). The neural control of bimanual movements in the elderly: Brain regions exhibiting age-related increases in activity, frequency-induced neural modulation, and task-specific compensatory recruitment. *Hum. Brain Mapp.* 31 1281–1295. 10.1002/hbm.20943 20082331 PMC6871108

[B53] Golestani N. Zatorre R. J. (2004). Learning new sounds of speech: Reallocation of neural substrates. *Neuroimage* 21 494–506. 10.1016/j.neuroimage.2003.09.071 14980552

[B54] Gratton C. Sun H. Petersen S. E. (2018). Control networks and hubs. *Psychophysiology* 55:e13032. 10.1111/psyp.13032 29193146 PMC5811327

[B55] Grefkes C. Geyer S. Schormann T. Roland P. Zilles K. (2001). Human somatosensory area 2: Observer-independent cytoarchitectonic mapping, interindividual variability, and population map. *Neuroimage* 14 617–631. 10.1006/nimg.2001.0858 11506535

[B56] Grodzinsky Y. (2000). The neurology of syntax: Language use without Broca’s area. *Behav. Brain Sci.* 23 1–21. 10.1017/s0140525x00002399 11303337

[B57] Grodzinsky Y. Friederici A. D. (2006). Neuroimaging of syntax and syntactic processing. *Curr. Opin. Neurobiol.* 16 240–246. 10.1016/j.conb.2006.03.007 16563739

[B58] Grodzinsky Y. Santi A. (2008). The battle for Broca’s region. *Trends Cogn. Sci.* 12 474–480. 10.1016/j.tics.2008.09.001 18930695

[B59] Grodzinsky Y. Deschamps I. Pieperhoff P. Iannilli F. Agmon G. Loewenstein Y. (2020). Logical negation mapped onto the brain. *Brain Struct. Funct.* 225 19–31. 10.1007/s00429-019-01975-w 31680213 PMC6957563

[B60] Hagoort P. (2014). Nodes and networks in the neural architecture for language: Broca’s region and beyond. *Curr. Opin. Neurobiol.* 28 136–141. 10.1016/j.conb.2014.07.013 25062474

[B61] Hagoort P. (2019). The neurobiology of language beyond single-word processing. *Science* 366 55–58. 10.1126/science.aax0289 31604301

[B62] Higo T. Mars R. B. Boorman E. D. Buch E. R. Rushworth M. F. S. (2011). Distributed and causal influence of frontal operculum in task control. *Proc. Natl. Acad. Sci. U.S.A.* 108 4230–4235. 10.1073/pnas.1013361108 21368109 PMC3054014

[B63] Holmes C. J. Hoge R. Collins L. Woods R. Toga A. W. Evans A. C. (1998). Enhancement of MR images using registration for signal averaging. *J. Comput. Assist. Tomogr.* 22 324–333. 10.1097/00004728-199803000-00032 9530404

[B64] Huschler M. A. Liem F. Jäncke L. Meyer M. (2013). Right and left perisylvian cortex and left inferior frontal cortex mediate sentence-level rhyme detection in spoken language as revealed by sparse fMRI. *Hum. Brain Mapp.* 34 3182–3192. 10.1002/hbm.22134 22711328 PMC6870020

[B65] Jung J. Cloutman L. L. Binney R. J. Lambon Ralph M. A. (2017). The structural connectivity of higher order association cortices reflects human functional brain networks. *Cortex* 97 221–239. 10.1016/j.cortex.2016.08.011 27692846 PMC5726605

[B66] Keller S. S. Crow T. Foundas A. Amunts K. Roberts N. (2009). Broca’s area: Nomenclature, anatomy, typology and asymmetry. *Brain Lang.* 109 29–48. 10.1016/j.bandl.2008.11.005 19155059

[B67] Kiwitz K. Brandstetter A. Schiffer C. Bludau S. Mohlberg H. Omidyeganeh M. (2022). Cytoarchitectonic maps of the human metathalamus in 3D space. *Front. Neuroanat.* 16:837485. 10.3389/fnana.2022.837485 35350721 PMC8957853

[B68] Kunert R. Willems R. M. Casasanto D. Patel A. D. Hagoort P. (2015). Music and language syntax interact in Broca’s area: An fMRI study. *PLoS One* 10:e0141069. 10.1371/journal.pone.0141069 26536026 PMC4633113

[B69] Laird A. R. Eickhoff S. B. Fox P. M. Uecker A. M. Ray K. L. Saenz J. J. Jr. (2011). The BrainMap strategy for standardization, sharing, and meta-analysis of neuroimaging data. *BMC Res. Notes* 4:349. 10.1186/1756-0500-4-349 21906305 PMC3180707

[B70] Laird A. R. Eickhoff S. B. Kurth F. Fox P. M. Uecker A. M. Turner J. A. (2009). ALE meta-analysis workflows via the BrainMap database: Progress towards a probabilistic functional brain atlas. *Front. Neuroinform.* 3:23. 10.3389/neuro.11.023.2009 19636392 PMC2715269

[B71] Laird A. R. Lancaster J. J. Fox P. T. (2005). BrainMap: The social evolution of a human brain mapping database. *Neuroinformatics* 3 65–77. 10.1385/ni:3:1:065 15897617

[B72] Levitin D. J. Menon V. (2003). Musical structure is processed in “language” areas of the brain: A possible role for Brodmann Area 47 in temporal coherence. *Neuroimage* 20 2142–2152. 10.1016/j.neuroimage.2003.08.016 14683718

[B73] Limb C. J. Kemeny S. Ortigoza E. B. Rouhani S. Braun A. R. (2006). Left hemispheric lateralization of brain activity during passive rhythm perception in musicians. *Anat. Rec. Pt. A Discov. Mol. Cell Evol. Biol.* 288A 382–389. 10.1002/ar.a.20298 16550585

[B74] Lorenz S. Weiner K. S. Caspers J. Mohlberg H. Schleicher A. Bludau S. (2017). Two new cytoarchitectonic areas on the human mid-fusiform gyrus. *Cereb. Cortex* 27 373–385. 10.1093/cercor/bhv225 26464475 PMC6248695

[B75] Lowell S. Y. Poletto C. J. Knorr-Chung B. R. Reynolds R. C. Simonyan K. Ludlow C. L. (2009). Sensory stimulation activates both motor and sensory components of the swallowing system. *Neuroimage* 42 285–295. 10.1016/j.neuroimage.2008.04.234 18515150 PMC2556067

[B76] Mahalanobis P. C. Majumdar D. N. Rao C. R. (1949). Anthropometric survey of the united provinces, 1941: A statistical study. *Sankhyâ Indian J. Stat.* 9 89–324.

[B77] Merker B. (1983). Silver staining of cell bodies by means of physical development. *J. Neurosci. Methods.* 9 235–241. 10.1016/0165-0270(83)90086-9 6198563

[B78] Morosan P. Schleicher A. Amunts K. Zilles K. (2005). Multimodal architectonic mapping of human superior temporal gyrus. *Anat. Embryol.* 210 401–406. 10.1007/s00429-005-0029-1 16170539

[B79] Moulier V. Mouras H. Pélégrini-Issac M. Glutron D. Rouxel R. Grandjean B. (2006). Neuroanatomical correlates of penile erection evoked by photographic stimuli in human males. *Neuroimage* 33 689–699. 10.1016/j.neuroimage.2006.06.037 16962339

[B80] Nichols T. Brett M. Andersson J. Wager T. Poline J.-B. (2005). Valid conjunction inference with the minimum statistic. *Neuroimage* 25 653–660. 10.1016/j.neuroimage.2004.12.005 15808966

[B81] Novick J. M. Trueswell J. C. Thompson-Schill S. L. (2005). Cognitive control and parsing: Reexamining the role of Broca’s area in sentence comprehension. *Cogn. Affect. Behav. Neurosci.* 5 263–281. 10.3758/cabn.5.3.263 16396089

[B82] Öngür D. Ferry A. T. Price J. L. (2003). Architectonic subdivision of the human orbital and medial prefrontal cortex. *J. Comp. Neurol.* 460 425–449. 10.1002/cne.10609 12692859

[B83] Palomero-Gallagher N. Eickhoff S. B. Hoffstaedter F. Schleicher A. Mohlberg H. Vogt B. A. (2015). Functional organization of human subgenual cortical areas: Relationship between architectonical segregation and connectional heterogeneity. *Neuroimage* 115 177–190. 10.1016/j.neuroimage.2015.04.053 25937490 PMC4801475

[B84] Petrides M. Pandya D. (1994). “Comparative architectonic analysis of the human and the macaque frontal cortex,” in *Handbook of neuropsychology*, eds Boller F. Grafman J. (Amsterdam: Elsevier), 17–58.

[B85] Price C. J. Friston K. J. (1997). Cognitive conjunction: A new approach to brain activation experiments. *Neuroimage* 5 261–270. 10.1006/nimg.1997.0269 9345555

[B86] Quabs J. Caspers S. Bludau S. Mohlberg H. Amunts K. (2021a). *Probabilistic cytoarchitectonic map of area Id4 (insula) (v5.1) [data set].* Jülich: EBRAINS. 10.25493/6495-07U

[B87] Quabs J. Caspers S. Bludau S. Mohlberg H. Amunts K. (2021b). *Probabilistic cytoarchitectonic map of area Id6 (insula) (v5.1) [data set].* Jülich: EBRAINS. 10.25493/PVEY-DPX

[B88] Quabs J. Caspers S. Schöne C. Mohlberg H. Bludau S. Dickscheid T. (2022). Cytoarchitecture, probability maps and segregation of the human insula. *Neuroimage* 260:119453. 10.1016/j.neuroimage.2022.119453 35809885

[B89] Quirmbach F. Limanowski J. (2022). A crucial role of the frontal operculum in task-set dependent visuomotor performance monitoring. *eNeuro.* 9, 1–12. 10.1523/eneuro.0524-21.2021 35165200 PMC8896555

[B90] Robinson J. L. Laird A. R. Glahn D. C. Lovallo W. R. Fox P. T. (2010). Metaanalytic connectivity modeling: Delineating the functional connectivity of the human amygdala. *Hum. Brain Mapp.* 31 173–184. 10.1002/hbm.20854 19603407 PMC2872058

[B91] Ruan J. Bludau S. Palomero-Gallagher N. Caspers S. Mohlberg H. Eickhoff S. B. (2018). Cytoarchitecture, probability maps, and functions of the human supplementary and pre-supplementary motor areas. *Brain Struct. Funct.* 223 4169–4186. 10.1007/s00429-018-1738-6 30187192 PMC6267244

[B92] Saal M. Bludau S. Mohlberg H. Caspers S. Amunts K. (2021a). *Probabilistic cytoarchitectonic map of area OP8 (frontal operculum) (v6.2) [data set].* Jülich: EBRAINS. 10.25493/1CTC-2GS

[B93] Saal M. Bludau S. Mohlberg H. Caspers S. Amunts K. (2021b). *Probabilistic cytoarchitectonic map of area OP9 (frontal operculum) (v6.2) [data set].* Jülich: EBRAINS. 10.25493/9TCJ-JZ3

[B94] Scheperjans F. Eickhoff S. B. Hömke L. Mohlberg H. Hermann K. Amunts K. (2008a). Probabilistic maps, morphometry, and variability of cytoarchitectonic areas in the human superior parietal cortex. *Cereb. Cortex* 18 2141–2157. 10.1093/cercor/bhm241 18245042 PMC3140197

[B95] Scheperjans F. Hermann K. Eickhoff S. B. Amunts K. Schleicher A. Zilles K. (2008b). Observer-independent cytoarchitectonic mapping of the human superior parietal cortex. *Cereb. Cortex* 18 846–867. 10.1093/cercor/bhm116 17644831

[B96] Schleicher A. Amunts K. Geyer S. Kowalski T. Schormann T. Palomero-Gallagher N. (2000). A stereological approach to human cortical architecture: Identification and delineation of cortical areas. *J. Chem. Neuroanat.* 20 31–47. 10.1016/s0891-0618(00)00076-4 11074342

[B97] Schleicher A. Amunts K. Geyer S. Morosan P. Zilles K. (1999). Observer-independent method for microstructural parcellation of cerebral cortex: A quantitative approach to cytoarchitectonics. *Neuroimage* 9 165–177. 10.1006/nimg.1998.0385 9918738

[B98] Schleicher A. Morosan P. Amunts K. Zilles K. (2009). Quantitative architectural analysis: A new approach to cortical mapping. *J. Autism Dev. Disord.* 39 1568–1581. 10.1007/s10803-009-0790-8 19582566

[B99] Schleicher A. Palomero-Gallagher N. Morosan P. Eickhoff S. B. Kowalski T. De Vos K. (2005). Quantitative architectural analysis: A new approach to cortical mapping. *Anat. Embryol.* 210 373–386. 10.1007/s00429-005-0028-2 16249867

[B100] Shaywitz B. A. Shaywltz S. E. Pugh K. R. Constable R. T. Skudlarski P. Fulbright R. K. (1995). Sex differences in the functional organization of the brain for language. *Nature* 373 607–609. 10.1038/373607a0 7854416

[B101] Sigl B. Caspers S. Bludau S. Mohlberg H. Eickhoff S. B. Amunts K. (2021a). *Probabilistic cytoarchitectonic map of area 6d1 (PreCG) (v7.1) [data set].* Jülich: EBRAINS. 10.25493/KSY8-H3F

[B102] Sigl B. Caspers S. Bludau S. Mohlberg H. Eickhoff S. B. Amunts K. (2021b). *Probabilistic cytoarchitectonic map of area 6d2 (PreCG) (v7.1) [data set].* Jülich: EBRAINS. 10.25493/WJQ5-HWC

[B103] Sigl B. Caspers S. Bludau S. Mohlberg H. Eickhoff S. B. Amunts K. (2021c). *Probabilistic cytoarchitectonic map of area 6d3 (SFS) (v7.1) [data set].* Jülich: EBRAINS. 10.25493/D41S-AG7

[B104] Skeide M. A. Brauer J. Friederici A. D. (2016). Brain functional and structural predictors of language performance. *Cereb. Cortex* 26 2127–2139. 10.1093/cercor/bhv042 25770126

[B105] Thompson-Schill S. L. D’Esposito M. Aguirre G. K. Farah M. J. (1997). Role of left inferior prefrontal cortex in retrieval of semantic knowledge: A reevaluation. *Proc. Natl. Acad. Sci. U.S.A.* 94 14792–14797. 10.1073/pnas.94.26.14792 9405692 PMC25116

[B106] Tillmann B. Janata P. Bharucha J. J. (2003). Activation of the inferior frontal cortex in musical priming. *Cogn. Brain Res.* 16 145–161. 10.1016/s0926-6410(02)00245-8 12668222

[B107] Tremblay P. Dick A. S. (2016). Broca and Wernicke are dead, or moving past the classic model of language neurobiology. *Brain Lang.* 162 60–71. 10.1016/j.bandl.2016.08.004 27584714

[B108] Unger N. Bludau S. Mohlberg H. Caspers S. Amunts K. (2021a). *Probabilistic cytoarchitectonic map of area OP5 (frontal operculum) (v3.2) [data set].* Jülich: EBRAINS. 10.25493/KN1A-YX4

[B109] Unger N. Bludau S. Mohlberg H. Caspers S. Amunts K. (2021b). *Probabilistic cytoarchitectonic map of area OP6 (frontal operculum) (v3.2) [data set].* Jülich: EBRAINS. 10.25493/RQKR-WE4

[B110] Unger N. Bludau S. Mohlberg H. Caspers S. Amunts K. (2021c). *Probabilistic cytoarchitectonic map of area OP7 (frontal operculum) (v3.2) [data set].* Jülich: EBRAINS. 10.25493/W2D1-DJF

[B111] Veldhuizen M. G. Albrecht J. Zelano C. Boesveldt S. Breslin P. Lundström J. N. (2011). Identification of human gustatory cortex by activation likelihood estimation. *Hum. Brain Mapp.* 32 2256–2266. 10.1002/hbm.21188 21305668 PMC3123671

[B112] Ventura-Campos N. Sanjuán A. González J. Palomar-García M.-Á. Rodríguez-Pujadas A. Sebastián-Gallés N. (2013). Spontaneous brain activity predicts learning ability of foreign sounds. *J. Neurosci.* 33 9295–9305. 10.1523/jneurosci.4655-12.2013 23719798 PMC6618579

[B113] von Economo C. Koskinas G. (1925). *Die Cytoarchitektonik der Hirnrinde des erwachsenen Menschen.* Wien: Springer Verlag.

[B114] Ward J. H. Jr. (1963). Hierarchical grouping to optimize an objective function. *J. Am. Stat. Assoc.* 58 236–244. 10.1080/01621459.1963.10500845

[B115] Ward N. S. Frackowiak R. S. J. (2003). Age-related changes in the neural correlates of motor performance. *Brain* 126 873–888. 10.1093/brain/awg071 12615645 PMC3717766

[B116] Wree A. Schleicher A. Zilles K. (1982). Estimation of volume fractions in nervous tissue with an image analyzer. *J. Neurosci. Methods* 6 29–43. 10.1016/0165-0270(82)90014-0 7121060

[B117] Zilles K. Amunts K. (2018). Cytoarchitectonic and receptorarchitectonic organization in Broca’s region and surrounding cortex. *Curr. Opin. Behav. Sci.* 21 93–105. 10.1016/j.cobeha.2018.02.011

[B118] Zysset S. Schroeter M. L. Neumann J. Von Cramon D. Y. (2007). Stroop interference, hemodynamic response and aging: An event-related fMRI study. *Neurobiol. Aging* 28 937–946. 10.1016/j.neurobiolaging.2006.05.008 21887888

