# Supplementary material for: Cytoarchitectonic mapping of the human frontal operculum—New correlates for a variety of brain functions
Source: Front Hum Neurosci. 2023 Jun 28;17:1087026. doi: 10.3389/fnhum.2023.1087026 (PMC10336231; doi:10.3389/fnhum.2023.1087026)
Supplement: Supplementary file 1 [file Data_Sheet_1.docx]

**SUPPLEMENTARY TABLE 1** Summary of the post mortem brains of five females (F) and five males (M) obtained for the cytoarchitectonic analysis of the human FOp.

| ***Brain*** | ***Age [years]*** | ***Gender*** | ***Brain weight (fresh) [g]*** | ***Cause of death*** |
| --- | --- | --- | --- | --- |
| BC4 | 75 | M | 1,349 | Toxic glomerulo-nephritis |
| BC5 | 59 | F | 1,142 | Cardiorespiratory insufficiency |
| BC6 | 54 | M | 1,622 | Myocardial infarction |
| BC7 | 37 | M | 1,437 | Right heart failure |
| BC8 | 72 | F | 1,216 | Renal failure |
| BC9 | 79 | F | 1,110 | Cardiorespiratory insufficiency |
| BC10 | 85 | F | 1,046 | Mesenteric artery infarction |
| BC12 | 43 | F | 1,198 | Pulmonary embolism |
| BC20 | 65 | M | 1,392 | Congestive heart failure, respiratory  failure |
| BC21 | 30 | M | 1,409 | Broncho-pneumonia, deep vein thrombosis |
|  | Ø: 59.9 years |  |  |  |

**SUPPLEMENTARY TABLE 2** Sleuth search results from the BrainMap database (Fox et al., 2002; Laird et al., 2005, available online, <http://brainmap.org>). Table including results of left, right, and merged maps. Merged maps led to a total number of 2,620 matching functional neuroimaging experiments with activations in the FOp region. Since the merged maps were generated by pooling of left and right proportions of the individual maps per area, the merged results differed from the sum of the maps per hemisphere.

| ***Area*** | ***Hemisphere*** | ***Found papers*** | ***Subjects*** | ***Experiments w/ matching criteria*** | ***Conditions*** | ***Locations*** |
| --- | --- | --- | --- | --- | --- | --- |
| **Op5** | Left | 84 | 1,228 | 92 | 218 | 1,365 |
|  | Right | 91 | 1,682 | 103 | 220 | 1,891 |
|  | Merged | 156 | 2,563 | 180 | 396 | 2,985 |
| **Op6** | Left | 243 | 3,749 | 299 | 644 | 5,683 |
|  | Right | 83 | 1,195 | 103 | 231 | 1,597 |
|  | Merged | 291 | 4,514 | 380 | 801 | 6,796 |
| **Op7** | Left | 41 | 596 | 42 | 100 | 810 |
|  | Right | 27 | 366 | 29 | 68 | 442 |
|  | Merged | 67 | 962 | 71 | 166 | 1,252 |

**SUPPLEMENTARY TABLE 3** Best matches of FOp areas with co-activational components of the Julich-Brain Atlas 2.9 calculated by meta-analytic connectivity modeling (MACM).

| ***Component*** | ***Best match*** | ***Correlation*** | ***Max p*** |
| --- | --- | --- | --- |
| ***Co-activations Op5 left*** | | | |
| 1 | Area Op5 (Frontal Operculum) left | 0.51 | 0.82 |
|  | Area Id4 (Insula) left | 0.39 | 0.75 |
|  | Area TE 1.2 (HESCHL) left | 0.32 | 0.72 |
|  | Area OP3 (POperc) left | 0.30 | 0.85 |
| 2 | Area 2 (PostCS) left | 0.32 | 0.84 |
|  | Area PFt (IPL) left | 0.29 | 0.85 |
|  | Area 3b (PostCG) left | 0.18 | 0.91 |
|  | Area 1 (PostCG) left | 0.01 | 0.95 |
| 3 | Area 4p (PreCG) left | 0.36 | 0.82 |
|  | Area 4a (PreCG) left | 0.25 | 0.93 |
|  | Area 3b (PostCG) left | 0.23 | 0.91 |
|  | Area 2 (PostCS) left | 0.08 | 0.84 |
| 4 | Temporal-to-Parietal (GapMap) left | -0.03 | 1.00 |
|  | CGM (Metathalamus) left | -0.04 | 1.00 |
|  | HC-Transsubiculum (Hippocampus) left | -0.05 | 0.79 |
|  | HC-Presubiculum (Hippocampus) left | -0.05 | 0.53 |
| 5 | Area 6mp (SMA, mesial SFG) left | 0.20 | 0.98 |
|  | Area 6ma (preSMA, mesial SFG) left | 0.15 | 1.00 |
|  | Area 6ma (preSMA, mesial SFG) right | 0.07 | 1.00 |
|  | Frontal-to-Occipital (GapMap) left | 0.04 | 1.00 |
| 6 | Area FG4 (FusG) right | -0.03 | 1.00 |
|  | Area FG3 (FusG) right | -0.04 | 0.91 |
|  | Area hOc3v (LingG) right | -0.04 | 0.68 |
|  | Area hOc2 (V2, 18) right | -0.04 | 0.90 |
| 7 | Area Op5 (Frontal Operculum) right | 0.44 | 0.73 |
|  | Area Id6 (Insula) right | 0.39 | 1.00 |
|  | Area Op8 (Frontal Operculum) right | 0.36 | 0.96 |
|  | Area Op7 (Frontal Operculum) right | 0.32 | 0.56 |
| ***Co-activations Op5 right*** | | | |
| 1 | Area Op5 (Frontal Operculum) left | 0.46 | 0.82 |
|  | Area OP1 (POperc) left | 0.36 | 0.89 |
|  | Area Id4 (Insula) left | 0.35 | 0.75 |
|  | Area Op6 (Frontal Operculum) left | 0.31 | 0.82 |
| 2 | Area 4p (PreCG) left | 0.36 | 0.82 |
|  | Area 4a (PreCG) left | 0.16 | 0.93 |
|  | Area 3b (PostCG) left | 0.14 | 0.91 |
|  | Area 3a (PostCG) left | 0.03 | 0.86 |
| 3 | Area FG4 (FusG) left | -0.04 | 1.00 |
|  | Area FG3 (FusG) left | -0.04 | 0.95 |
|  | Area FG2 (FusG) left | -0.05 | 0.87 |
|  | Area FG1 (FusG) left | -0.06 | 0.85 |
| 4 | Frontal-to-Temporal-II (GapMap) right | -0.04 | 1.00 |
|  | CGM (Metathalamus) left | -0.05 | 1.00 |
|  | BST (Bed Nucleus) left | -0.05 | 0.89 |
|  | CGM (Metathalamus) right | -0.05 | 0.98 |
| 5 | Area 6ma (preSMA, mesial SFG) right | 0.29 | 1.00 |
|  | Area 6mp (SMA, mesial SFG) right | 0.20 | 1.00 |
|  | Area 6mp (SMA, mesial SFG) left | 0.13 | 0.98 |
|  | Area 6ma (preSMA, mesial SFG) left | 0.07 | 1.00 |
| 6 | Area Op5 (Frontal Operculum) right | 0.60 | 0.73 |
|  | Area Op6 (Frontal Operculum) right | 0.40 | 0.64 |
|  | Area Id4 (Insula) right | 0.34 | 0.66 |
|  | Area OP4 (POperc) right | 0.31 | 0.83 |
|  | Area OP3 (POperc) right | 0.30 | 0.68 |
| ***Co-activations Op6 left*** | | | |
| 1 | Area 44 (IFG) left | 0.30 | 1.00 |
|  | Area Op6 (Frontal Operculum) left | 0.29 | 0.82 |
|  | Area Id6 (Insula) left | 0.25 | 0.97 |
|  | Area Op6 (Frontal Operculum) right | 0.24 | 0.64 |
| 2 | Area FG2 (FusG) left | 0.22 | 0.87 |
|  | Area FG4 (FusG) left | 0.07 | 1.00 |
|  | Area FG1 (FusG) left | 0.02 | 0.85 |
|  | Temporal-to-Parietal (GapMap) left | -0.02 | 1.00 |
| 3 | Area 6ma (preSMA, mesial SFG) right | 0.43 | 1.00 |
|  | Area 6ma (preSMA, mesial SFG) left | 0.37 | 1.00 |
|  | Frontal-to-Occipital (GapMap) left | 0.04 | 1.00 |
|  | Frontal-to-Occipital (GapMap) right | 0.04 | 1.00 |
| 4 | Area FG4 (FusG) right | -0.06 | 1.00 |
|  | Area hOc3v (LingG) right | -0.06 | 0.68 |
|  | Area hOc2 (V2, 18) right | -0.06 | 0.90 |
|  | Area FG3 (FusG) right | -0.07 | 0.91 |
| 5 | Area hIP2 (IPS) right | 0.48 | 0.78 |
|  | Area hIP3 (IPS) right | 0.19 | 0.92 |
|  | Area hIP1 (IPS) right | 0.11 | 1.00 |
|  | Area PFm (IPL) right | 0.04 | 0.99 |

| ***Co-activations Op6 right*** | | | |
| --- | --- | --- | --- |
| 1 | Area Op6 (Frontal Operculum) left | 0.48 | 0.82 |
|  | Area OP1 (POperc) left | 0.32 | 0.89 |
|  | Area TE 1.2 (HESCHL) left | 0.29 | 0.72 |
|  | Area Id6 (Insula) left | 0.28 | 0.97 |
| 2 | Area 6ma (preSMA, mesial SFG) right | 0.28 | 1.00 |
|  | Area 6ma (preSMA, mesial SFG) left | 0.13 | 1.00 |
|  | Frontal-to-Occipital (GapMap) right | 0.05 | 1.00 |
|  | Area 6mp (SMA, mesial SFG) right | 0.04 | 1.00 |
| 3 | CGM (Metathalamus) right | -0.06 | 0.98 |
|  | BST (Bed Nucleus) right | -0.11 | 1.00 |
| 4 | Area Op6 (Frontal Operculum) right | 0.62 | 0.64 |
|  | Area Op8 (Frontal Operculum) right | 0.33 | 0.96 |
|  | Area TE 1.2 (HESCHL) right | 0.33 | 0.56 |
|  | Area OP4 (POperc) right | 0.29 | 0.83 |
| 5 | Area TPJ (STG/SMG) right | 0.53 | 0.71 |
|  | Area PFcm (IPL) right | 0.45 | 0.88 |
|  | Area TE 2.2 (STG) right | 0.36 | 0.98 |
|  | Area PF (IPL) right | 0.21 | 0.81 |
| ***Co-activations Op7 left*** | | | |
| 1 | Area Op7 (Frontal Operculum) left | 0.64 | 0.66 |
|  | Area Id6 (Insula) left | 0.42 | 0.97 |
|  | Area Op8 (Frontal Operculum) left | 0.38 | 0.95 |
|  | Area Op6 (Frontal Operculum) left | 0.26 | 0.82 |
| 2 | Area 6ma (preSMA, mesial SFG) right | 0.39 | 1.00 |
|  | Frontal-to-Occipital (GapMap) right | 0.09 | 1.00 |
|  | Area 33 (ACC) right | 0.03 | 0.81 |
|  | Frontal-to-Occipital (GapMap) left | -0.00 | 1.00 |
| 3 | Area Op8 (Frontal Operculum) right | 0.53 | 0.96 |
|  | Area Op7 (Frontal Operculum) right | 0.48 | 0.56 |
|  | Area Id6 (Insula) right | 0.45 | 1.00 |
|  | Area Id7 (Insula) right | 0.16 | 1.00 |
| ***Co-activations Op7 right*** | | | |
| 1 | Area Id6 (Insula) left | 0.59 | 0.97 |
|  | Area Op8 (Frontal Operculum) left | 0.47 | 0.95 |
|  | Area Op7 (Frontal Operculum) left | 0.47 | 0.66 |
|  | Area Id7 (Insula) left | 0.43 | 1.00 |
| 2 | Area IFJ2 (IFS,PreCS) left | 0.29 | 0.81 |
|  | Frontal-II (GapMap) left | 0.08 | 1.00 |
|  | Area 44 (IFG) left | 0.02 | 1.00 |
|  | Area 4p (PreCG) left | -0.02 | 0.82 |
| 3 | Area hOc4v (LingG) left | -0.04 | 0.98 |
|  | Area FG4 (FusG) left | -0.04 | 1.00 |
|  | Area FG3 (FusG) left | -0.04 | 0.95 |
|  | Area FG2 (FusG) left | -0.05 | 0.87 |
| 4 | Frontal-to-Temporal-II (GapMap) right | -0.03 | 1.00 |
|  | CGM (Metathalamus) right | -0.04 | 0.98 |
|  | CGM (Metathalamus) left | -0.04 | 1.00 |
|  | CA3 (Hippocampus) right | -0.05 | 0.50 |
| 5 | Area 6ma (preSMA, mesial SFG) right | 0.21 | 1.00 |
|  | Area 6ma (preSMA, mesial SFG) left | 0.18 | 1.00 |
|  | Frontal-to-Occipital (GapMap) right | 0.05 | 1.00 |
|  | Frontal-to-Occipital (GapMap) left | 0.04 | 1.00 |
| 6 | Area Op8 (Frontal Operculum) right | 0.60 | 0.96 |
|  | Area Op7 (Frontal Operculum) right | 0.57 | 0.56 |
|  | Area Id6 (Insula) right | 0.56 | 1.00 |
|  | Area Id7 (Insula) right | 0.27 | 1.00 |
| 7 | Area hIP3 (IPS) right | 0.37 | 0.92 |
|  | Area hIP1 (IPS) right | 0.14 | 1.00 |
|  | Area hIP6 (IPS) right | 0.07 | 0.96 |
|  | Temporal-to-Parietal (GapMap) right | -0.01 | 1.00 |
| 8 | Area TE 2.2 (STG) right | 0.45 | 0.98 |
|  | Area OP1 (POperc) right | 0.12 | 0.85 |
|  | Area PFop (IPL) right | 0.06 | 0.74 |
|  | Area PFcm (IPL) right | 0.04 | 0.88 |

**SUPPLEMENTARY TABLE 4** Summary containing co-activated cortical areas of the Julich-Brain Atlas calculated by meta-analytic connectivity modeling (MACM) in combination with their reported functions. For an overview of previous cytoarchitectonic studies of our institute, see EBRAINS research infrastructure of the Human Brain Project (<https://www.humanbrainproject.eu/en/explore-the-brain/>).

| ***Co-activated area/nucleus*** | ***Function(s)*** | ***Reference(s) from Julich-Brain Atlas (Amunts et al., 2020)*** |
| --- | --- | --- |
| 1 | Primary somatosensory cortex | Geyer et al., 1999, 2000 |
| 2 | Registration of shape and curvature changes; higher level of somatosensory processing | Grefkes et al., 2001 |
| 3a | Primary somatosensory cortex | Geyer et al., 1999, 2000 |
| 3b |  | Geyer et al., 1999, 2000 |
| 33 | Processing of painful stimuli | Palomero-Gallagher et al., 2015 |
| 44 | Language production and perception; phonological and syntactical processing; verbal fluency; spontaneous speech; semantic processing; word production | Amunts et al., 1999, 2004 |
| 4a | Primary motor cortex: dual representation of the fingers | Geyer et al., 1996 |
| 4p | Motor functions; learning; cognitive processes; perception | Geyer et al., 1996 |
| 6ma | Motor functions;  learning; cognitive processes; perception | Ruan et al., 2018 |
| 6mp | Motor functions | Ruan et al., 2018 |
| BST (Bed Nucleus) | Stress processing; certain forms of anxiety | Brandstetter et al., 2021 |
| CA3 (Hippocampus) | Memory and learning | Palomero-Gallagher et al., 2020 |
| CGM (Metathalamus) | Processing visual and auditory information | Kiwitz et al., 2022 |
| FG1 | Ventral visual cortex:  Identiﬁcation of visual objects, faces, and word forms;  scene processing  (Lorenz et al., 2017) | Caspers et al., 2013 |
| FG2 | Face and word processing | Caspers et al., 2013 |
| FG3 | Scene processing | Lorenz et al., 2017 |
| FG4 | Face and word processing | Lorenz et al., 2017 |
| HC-Presubiculum (Hippocampus) | Memory and learning | Palomero-Gallagher et al., 2020 |
| HC-Transsubiculum (Hippocampus) |  | Palomero-Gallagher et al., 2020 |
| hIP1 (IPS) | Numerical processing; visuo-spatial and visuo-motor operations; spatial and object working memory | Choi et al., 2006 |
| hIP2 (IPS) | Numerical processing; calculation | Choi et al., 2006 |
| hIP3 (IPS) | Visuo-motor integration; reaching movements; calculation | Scheperjans et al., 2008a, 2008b |
| hIP6 (IPS) | Action inhibition; language/orthography; reasoning | Richter et al., 2018 |

| hOc2 | Human visual cortex | Amunts et al., 2021 |
| --- | --- | --- |
| hOc3v | Ventral extrastriate human visual cortex | Rottschy et al., 2007 |
| hOc4v |  | Rottschy et al., 2007 |
| Id4 | Motor functions | Quabs et al., 2022 |
| Id6 | Socio-emotional processing and cognitive functions | Quabs et al., 2022 |
| Id7 |  | Grodzinsky et al., 2020 |
| IFJ2 (IFS) | Working memory, language-related  processes, music processing, attention | Ruland et al., 2022 |
| OP1 | Anatomical correlate of the (functionally defined) human SII region ‘secondary somatosensory area’:  Somatosensory stimuli; pain-related activity;  somesthesis (Eickhoff et al., 2010) | Eickhoff et al., 2006a, 2006c |
| OP3 | Anatomical correlate of the (functionally defined) human SII region ‘secondary somatosensory area’:  Somatosensory stimuli | Eickhoff et al., 2006a, 2006c |
| OP4 | Anatomical correlate of the (functionally defined) human SII region ‘secondary somatosensory area’:  Somatosensory stimuli;  somesthesis (Eickhoff et al., 2010) | Eickhoff et al., 2006a, 2006c |
| Op8 | Language function | Saal et al., 2021a |
| PF (IPL) | Integration of information from different sensory modalities; wide range of higher cognitive functions, e.g. spatial attention, motor planning, and language-related tasks | Caspers et al., 2006, 2008 |
| PFcm (IPL) |  | Caspers et al., 2006, 2008 |
| PFm (IPL) |  | Caspers et al., 2006, 2008 |
| PFop (IPL) |  | Caspers et al., 2006, 2008 |
| PFt (IPL) |  | Caspers et al., 2006, 2008 |
| TE 1.2 | Primary auditory cortex | Morosan et al., 2001; Rademacher et al., 2001 |
| TE 2.2 |  | Morosan et al., 2005 |
| TPJ | Attention; social interaction (Krall et al., 2014) | Zachlod et al., 2021 |

**Abbreviations:**

BST = bed nucleus of the stria terminalis; CGM = medial geniculate body; IFS = inferior frontal sulcus; IPL = inferior parietal lobule; IPS = intraparietal sulcus; TPJ = temporoparietal junction

**Additional references from Supplementary Table 4 (and Figure 10):**

Amunts, K., Malikovic, A., Mohlberg, H., Schormann, T., & Zilles, K. (2021). Probabilistic cytoarchitectonic map of Area hOc2 (V2, 18) (v4.2) [Data set]. EBRAINS. DOI: 10.25493/GR0Z-E15

Brandstetter, A., Mohlberg, H., Bludau, S., Evans, A. C., & Amunts, K. (2021). Probabilistic cytoarchitectonic map of bed nucleus of the stria terminalis (BST, basal forebrain) (v6.1) [Data set]. EBRAINS.

Eickhoff, S. B., Jbabdi, S., Caspers, S., Laird, A. R., Fox, P. T., Zilles, K., & Behrens, T. E. J. (2010). Anatomical and functional connectivity of cytoarchitectonic areas within the human parietal operculum. *Journal of Neuroscience, 30*(18), 6409–6421. https://doi.org/10.1523/JNEUROSCI.5664-09.2010

Krall, S. C., Rottschy, C., Oberwelland, E., Bzdok, D., Fox, P. T., Eickhoff, S. B., Fink, G. R., & Konrad, K. (2014). The role of the right temporoparietal junction in attention and social interaction as revealed by ALE meta-analysis. *Brain Structure and Function, 220,* 587–604. https://doi.org/10.1007/s00429-014-0803-z

Kurth, F., Eickhoff, S. B., Schleicher, A., Hoemke, L., Zilles, K., & Amunts, K. (2010). Cytoarchitecture and probabilistic maps of the human posterior insular cortex. *Cerebral Cortex, 20*(6), 1448–1461. https://10.1093/cercor/bhp208

Morosan, P., Rademacher, J., Schleicher, A., Amunts, K., Schormann, T., & Zilles, K. (2001). Human primary auditory cortex: Cytoarchitectonic subdivisions and mapping into a spatial reference system. *NeuroImage, 13*(4), 684–701. https://doi.org/10.1006/nimg.2000.0715

Palomero-Gallagher, N., Kedo, O., Mohlberg, H., Zilles, K., & Amunts, K. (2020). Multimodal mapping and analysis of the cyto- and receptorarchitecture of the human hippocampus. *Brain Structure and Function, 225*(3), 881–907. https://doi.org/10.1007/s00429-019-02022-4

Rademacher, J., Morosan, P., Schormann, T., Schleicher, A., Werner, C., Freund, H.-J., & Zilles, K. (2001). Probabilistic mapping and volume measurement of human primary auditory cortex. *NeuroImage, 13*(4), 669–683. https://doi.org/10.1006/nimg.2000.0714

Richter, M., Amunts, K., Mohlberg, H., Bludau, S., Eickhof, S. B., Zilles, K., & Caspers, S. (2019). Cytoarchitectonic segregation of human posterior intraparietal and adjacent parieto-occipital sulcus and its relation to visuomotor and cognitive functions. *Cerebral Cortex, 29*(3), 1305–1327. https://doi.org/10.1093/cercor/bhy245

Rottschy, C., Eickhoff, S., B., Schleicher, A., Mohlberg, H., Kujovic, M., Zilles, K., & Amunts, K. (2007). Ventral visual cortex in humans: cytoarchitectonic mapping of two extrastriate areas. *Human Brain Mapping, 28*(10), 1045–1059. https://doi.org/10.1002/hbm.20348

Ruland, S. H., Palomero-Gallagher, N., Hoffsteadter, F., Eickhoff, S. B., Mohlberg, H., & Amunts, K. (2022). The inferior frontal sulcus: Cortical segregation, molecular architecture and function. *Cortex, 153,* 235–256. https://doi.org/10.1016/j.cortex.2022.03.019

Zachlod, D., Mohlberg, H. & Amunts, K. (2021). Probabilistic cytoarchitectonic map of Area TPJ (STG/SMG) (v6.2) [Data set]. EBRAINS.
